# Evaluation of a novel chemiluminescent immunoassay for autoantibody detection in pemphigus and bullous pemphigoid

**DOI:** 10.3389/fimmu.2025.1576635

**Published:** 2025-06-30

**Authors:** Na Zhang, Ming Wang, Lili Hou, Yanyu Zhang, Yan Zhao, Luyang Du, Xiaoqing Li, Xia Liu, Hongwei Zhou, Jiaxuan Hao, Jing Tian, Qiuping Yu, Lixia Li, Fangming Cheng, Hai-Zhou Zhou

**Affiliations:** ^1^ Department of Laboratory Diagnostics, The First Affiliated Hospital of Harbin Medical University, Harbin, China; ^2^ Global Academic Department, Shenzhen YHLO Biotech Co., Ltd, Shenzhen, Guangdong, China; ^3^ Dermatology, The First Affiliated Hospital of Harbin Medical University, Harbin, China; ^4^ Reagent R&D Center, Shenzhen YHLO Biotech Co., Ltd, Shenzhen, Guangdong, China

**Keywords:** pemphigus, bullous pemphigoid, desmoglein, BP180, BP230, chemiluminescent immunoassay

## Abstract

**Background:**

A novel fully automated chemiluminescent immunoassay (CLIA) has been developed to quantify autoantibodies specific for desmogleins (Dsg1, Dsg3), BP180, and BP230, key target antigens in pemphigus and bullous pemphigoid. This study aims to evaluate the diagnostic accuracy of the CLIA in diagnosing pemphigus and BP and its correlation with disease severity and clinical features.

**Methods:**

Sera from 106 bullous pemphigoid and 54 pemphigus patients and control sera from 153 patients with other skin disease and 121 healthy volunteers were included. CLIA and BIOCHIP mosaic-based indirect immunofluorescence (IIFT-BIOCHIP) were performed for all bullous pemphigoid and pemphigus patients, with ELISA conducted for most. Disease severity was assessed using the Body Surface Area scoring.

**Results:**

This CLIA showed strong agreement with IIFT-BIOCHIP, achieving area under the curve (AUC) values of 0.92 for anti-Dsg1/anti-Dsg3 and 0.84 for anti-BP180/anti-BP230 for differentiating pemphigus and BP. It outperformed ELISA (AUC: 0.73, 0.75) and was comparable to IIFT-BIOCHIP (AUC: 0.93, 0.87). The assay showed superior detection range and sensitivity compared to ELISA. Autoantibody levels correlated with disease severity and clinical symptoms, with elevated levels of anti-Dsg3 associated with mucosal lesions, anti-Dsg1/anti-Dsg3 associated with Nikolsky sign, and elevated anti-BP180/anti-BP230 levels linked to pruritus.

**Conclusions:**

The novel CLIA, the first to quantify four major autoimmune blister disease autoantibodies (anti-Dsg1/anti-Dsg3/anti-BP180/anti-BP230) using a single sample tube, offers a simple and time-efficient test for diagnosing and screening pemphigus and BP. Its ability to assess disease severity and clinical relevance makes it a valuable tool for managing these conditions.

## 1 Introduction

Autoimmune bullous diseases (AIBDs), including bullous pemphigoid (BP) and pemphigus, are rare but potentially life-threatening skin disorders (1). Both diseases are characterized by autoantibodies targeting critical structural proteins (2). In BP, autoantibodies attack BP180 and BP230, components of hemidesmosomes that maintain dermo-epidermal junction. In pemphigus, autoantibodies target Desmoglein 1 (Dsg1) and 3 (Dsg3), desmosomal cadherins that link adjacent keratinocytes. Disruption of these proteins leads to blister formation and acantholysis. Pemphigus vulgaris (PV) and pemphigus foliaceus (PF) are the two most common types of pemphigus. Typically, the cutaneous mucocutaneous type of PV can be detected as positive for both anti-Dsg3 and anti-Dsg1 antibodies, the mucocutaneous-dominant type of PV can be detected as positive for anti-Dsg3 antibodies only, while only anti-Dsg1 antibodies can be detected as positive in PF (3).

The psychological impact of AIBDs is significant, with many patients suffering from anxiety and depression, especially in the early stages (4). If left untreated, both conditions can be fatal within three years due to complications such as protein and fluid loss, infections, and sepsis (5). Early diagnosis and treatment are essential to improve prognosis and quality of life (6, 7).

However, because of their rarity and varied clinical presentations, diagnosis can be delayed. BP may begin with a nonbullous phase, causing mild to severe pruritus, sometimes with eczematous, papular, or urticarial skin lesions that last for weeks or months. In most cases (80%), it progresses to the bullous stage, with fluid-filled blisters, erythema, and often affects the flexural areas and lower trunk, sometimes with mucosal involvement (8, 9). In contrast, pemphigus is characterized by intraepidermal acantholysis and a positive Nikolsky sign (9). Pemphigus vulgaris (PV) primarily presents with mucosal erosions, while pemphigus foliaceus (PF) causes superficial, fragile skin blisters without mucosal involvement (10). Despite these distinctive features, there are clinical overlap between the two diseases, along with significant heterogeneity within each, complicating diagnosis (9).

The diagnosis of BP and pemphigus involves a multi-step process, beginning with clinical evaluation, followed by histopathology, and then various immunological tests to identify target antigens and differentiate between subtypes (6, 7). Serological detection of autoantibodies (Dsg1/3, BP180/230) aids in diagnosis and subclassification, with common methods including indirect immunofluorescence (IIF), enzyme-linked immunosorbent assay (ELISA), and chemiluminescent assay (CLIA). The IIF method used in this study is BIOCHIP mosaic-based indirect immunofluorescence (IIFT-BIOCHIP). IIFT-BIOCHIP method utilizes the biochip mosaic technology, which enables the simultaneous detection of multiple different autoantibodies, thereby achieving comprehensive screening and confirmation in a single testing process. This approach significantly facilitates the differential diagnosis of AIBDs (11). Although ELISA and IIFT-BIOCHIP exhibit comparable diagnostic accuracy for pemphigus and BP, IIFT-BIOCHIP, as a semi-quantitative tool, cannot accurately monitor antibody levels, and the provided interpretations are subjective, which limits its clinical application value (12–14). ELISA provides high specificity and sensitivity, as well as high-throughput and quantitative detection, but it is sensitive to operational conditions, may have cross-reactivity, a limited detection range, a high cost, and high sample quality requirements, and cannot directly detect antigen-antibody complexes (15).

CLIA is an analytical technique that combines highly specific immune reactions with highly sensitive chemiluminescence detection. Direct chemiluminescent labels participate directly in luminescent reactions without enzymatic catalysis during immunoassays. These labels possess specific structural groups that generate luminescence and can be conjugated directly to antigens or antibodies. This study employs magnetic particle-based acridinium ester direct chemiluminescence technology. The principle involves conjugating acridinium ester to polymer chains on the surface of magnetic particles, forming acridinium ester-modified magnetic particles. When these modified particles react with specific oxidants (e.g., alkaline hydrogen peroxide), the acridinium ester undergoes hydrolysis and decomposition under alkaline conditions, leading to chemiluminescence emission. Quantitative analysis of target analytes is achieved by measuring the intensity of the emitted luminescence signal (16). CLIA demonstrates higher sensitivity and a broader detection range, enabling precise antibody quantification to monitor disease activity. Its streamlined, automatable workflow enhances operational efficiency, and superior cost-effectiveness in equipment/reagent utilization makes it ideal for high-throughput clinical testing. Further comparisons of these methodologies remain critical to optimizing AIBD diagnostic strategies. Multiplex detection capabilities and excellent reproducibility further enhance their clinical utility (6, 7, 13, 15).

To address the need for timely and accurate diagnosis, a novel CLIA for diagnosing AIBDs was developed. This assay simultaneously measures four key autoantibodies—anti-Dsg1, anti-Dsg3, anti-BP180, and anti-BP230—using a single serum tube on a fully automated iFlash platform, enabling the differentiation of various AIBD subtypes. In clinical settings where prompt treatment is often critical, this assay offers a rapid and reliable diagnostic tool.

To our knowledge, this study represents the first systematic comparison of IIFT-BIOCHIP, ELISA, and CLIA for antibody detection in AIBDs. We conducted a comparative analysis to evaluate the detection performance of these methods in AIBD patients, individuals with diseases easily confused with AIBDs (such as psoriasis, eczema, and herpes zoster), and healthy controls. The CLIA underwent performance evaluation, with reference ranges established and validated in a healthy population, along with cutoff values for AIBD diagnosis. Furthermore, we assessed the diagnostic accuracy of the novel CLIA compared with IIFT-BIOCHIP and ELISA in cases of pemphigus and BP. Additionally, we examined the correlation between autoantibody levels, disease severity, and clinical features, offering comprehensive insights into its clinical utility.

## 2 Materials and equipments

### 2.1 Human participates and samples

This was a single-center retrospective study. Sera were collected in Department of Dermatology or Department of Laboratory Diagnostics of First Affiliated Hospital of Harbin Medical University between 2022 and 2024.

In the performance evaluation section, this study used samples from patients with pemphigus foliatus (150 cases), pemphigus vulgaris (54 cases), bullous pemphigoid (201 cases) and bullous pemphigoid (200 cases) (confirmed positive by marketed products of the same type), and samples from the healthy population (150, 201, 202, and 200 cases, respectively) to determine the positive judgment values (cut off values) of the CLIA for anti-Dsg1, Dsg3, BP180, and BP230 antibodies. The cut off values of anti-Dsg1, Dsg3, BP180, and BP230 antibodies were confirmed by using 100, 40, 100, 100, 100 samples (50/50 for patient samples and healthy population samples, respectively).

**Figure 1:**
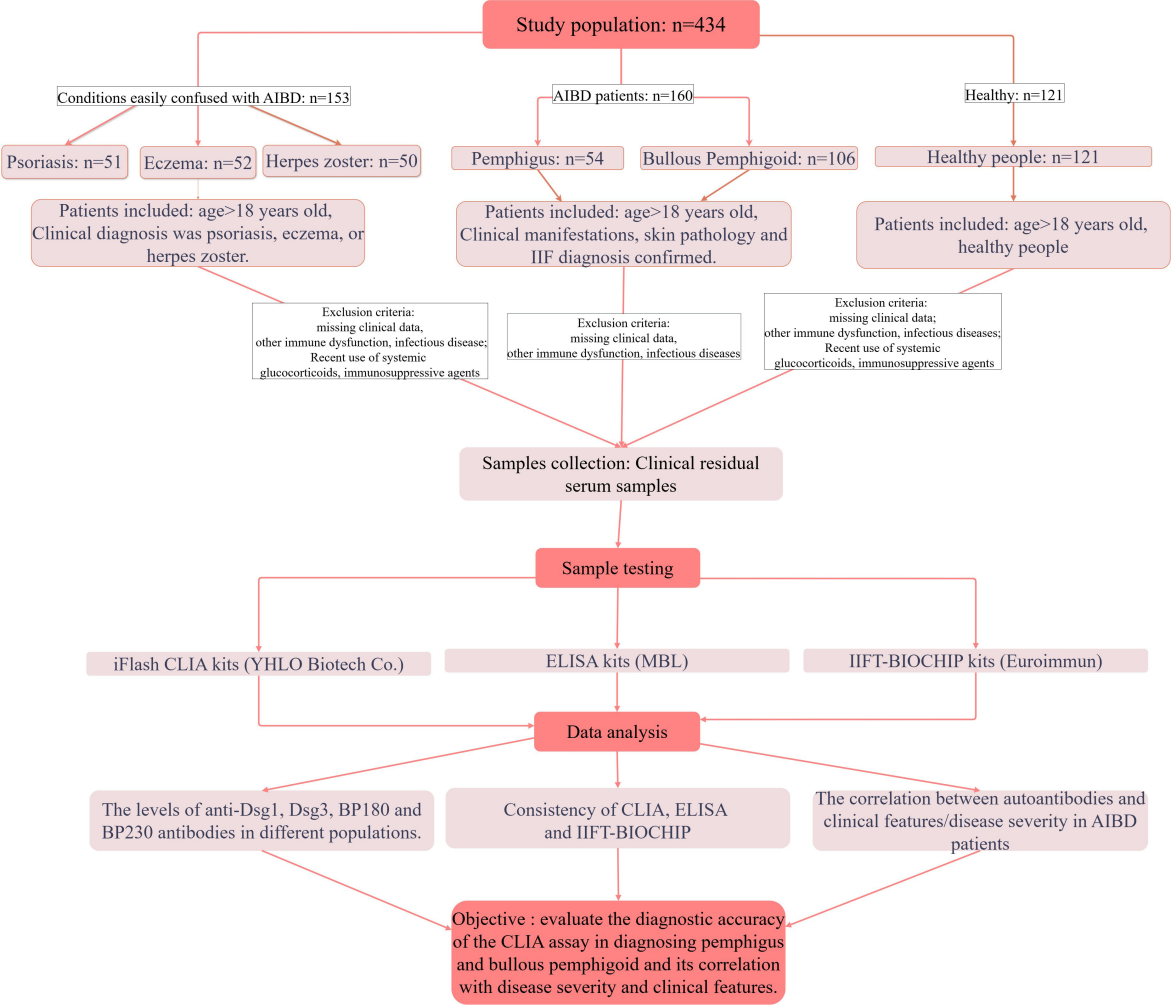
Flow chart of this study.

In the diagnostic performance assessment and methodology comparison section, 434 consecutive patients were enrolled in this study, with 160 AIBDs (54 pemphigus and 106 BP), 153 diagnosed with conditions easily confused with AIBD (52 eczema, 50 herpes zoster, 51 psoriasis) and 121 healthy people. The inclusion and exclusion criteria were outlined in the flow chart (**Figure 1**). The sample size was calculated using the parameter estimation method. Patients with AIBDs were diagnosed by combining clinical presentation, IIF and/or histopathology.

This study was approved by the Ethics Committee of First Affiliated Hospital of Harbin Medical University (No. 2024248) and was conducted in line with the principles of the Declaration of Helsinki. All samples were used after obtaining informed consent.

Instruments and reagents used in this study are described in **Supplementary Table 1**.

## 3 Methods

### 3.1 Performance evaluation of CLIA

#### 3.1.1 Evaluation of the precision of CLIA

The precision study referred to the method of EP05-A3 used a multifactorial integrated nested design (3*20*2*2) for the experiment. That is, antibody-negative, weak-positive, and positive samples (Serum 1~3) for each of four AIBD antibodies were tested in three different laboratories by three different operators on three instruments with the same batch number of reagents, and two rounds of testing were conducted for each sample per day (with at least 2 hours interval between each round of testing), with two replicates for each test, for a total of 20 days. If the CV of Repeatability is ≤10.0% and the CV of Reproducibility is ≤15.0%, the requirement is satisfied.

#### 3.1.2 Evaluation of the anti-interference ability of CLIA

The test of anti-interference ability referred to relevant standards and guidance documents such as EP7-A3 Interference Testing in Clinical Chemistry issued by the American Clinical and Laboratory Standardization Institute (CLSI) and China’s Guidelines for Interference Experiments (WS/T 416-2013), and assessed the effects of endogenous interfering substances on the assay, such as hemoglobin, bilirubin, triglycerides, total serum protein, rheumatoid factor (RF), human anti-mouse antibody (HAMA), antinuclear antibody (ANA) and other endogenous interfering substances on the assay were evaluated. If the relative deviation (dobs) is within ±10.0%, it can be judged that the deviation caused by the evaluated interfering substance does not exceed the permissible standard, and the substance is not considered to be an interfering substance.

#### 3.1.3 Performance characteristics of CLIA

The Limit of blank (LoB), Limit of detection (LoD) and Limit of quantitation (LoQ) values for the four reagents of the CLIA have been verified. LoB represents the maximum analyte concentration statistically expected from repeated measurements of a blank (analyte-free) sample, defining the threshold above on which a signal cannot be attributed to background noise. LoD means the lowest concentration of an analyte that can be reliably distinguished from the LoB with a high degree of confidence, and is the concentration limit at which detection is feasible, as determined by using the measured LOB and test replicates of samples known to contain low concentrations of the analyte. LoQ is the lowest concentration of an analyte that can be reliably detected while meeting certain predetermined target values for bias and inaccuracy. LoB, LoD, and LoQ of CLIA reagents were determined with reference to the document EP17-A2 Evaluation of Detection Capability for Clinical Laboratory Measurement Procedures by CLSI, and the experimental methods in the relevant industry standards for chemiluminescent reagent products. For the LoB verification, two blank samples were measured in four replicates each day for three days. For the LoD verification, two low level samples at the LoD claim measurand cencentration were measured in four replicates each day for the same three days. For the LoQ verification, two samples at the LoQ claim measured concentration were measured in five replicates each day for three days.

#### 3.1.4 Evaluation of the linear range of the CLIA

Verify the linearity of the CLIA reagent by referring to the document EP06-A. Two serum samples with low and high concentrations added (starting serum) were prepared separately, mixed and diluted into 9 medium concentration samples with different dilution ratios. All nine linear dilutions and starting serum samples were tested simultaneously, with three replicates per sample, and the assay data were analyzed by regression.

#### 3.1.5 Establishment and evaluation of cutoff values for the CLIA

This part of the study was based on the Receiver Operating Characteristic curve (ROC) method recommended by CLSI EP24-A2 Assessment of the Diagnostic Accuracy of Laboratory Tests Using Receiver Operating Characteristic Curves and the relevant registration technical guidelines. The ROC curve method was used to establish the positive judgment value, and the statistical software SPSS 22.0 was used to analyze the specificity and sensitivity of the concentrations of the patient samples and the samples from the healthy population by using the ROC curve, and the concentration corresponding to the most suitable specificity and sensitivity was selected as the positive judgment value.

### 3.2 CLIA for anti-Dsg1, anti-Dsg3, anti-BP180, anti-BP230

Antibody titers against Dsg1, Dsg3, BP180, and BP230 were measured using the chemiluminescent immunoassay (CLIA) method, which is fully automated and integrated with the iFlash system (Shenzhen YHLO Biotechnology Co., Ltd., Shenzhen, China). Each recombinant protein for CLIA was prepared similarly to ELISA, i.e., recombinant extracellular domains of Dsg1 and Dsg3 produced by CHO cells, and a recombinant NC16a domain of BP180 produced as a fusion protein in E. coli. For BP230, a recombinant protein produced in E. coli was used. Serum samples were reacted with magnetic beads coated with recombinant Dsg1, Dsg3, BP180, or BP230 proteins. Next, the immunocomplexes were reacted with an acridinium-labeled mouse anti-human IgG antibody. After the addition of the pre-trigger and trigger solutions, a chemiluminescent reaction commenced. A photomultiplier was used as the detector, which increased the dynamic range.

The experiment was performed according to manufacturer’s instructions. First, isolate the serum by centrifuging the blood sample at 3000 rpm for 10 minutes. Before use, all reagents must be equilibrated to room temperature (18~25°C) and the magnetic microbeads should be well mixed. A 3-point calibration is then performed using the provided calibrators. For sample loading, 10μL of the prepared sample is introduced into the instrument, and then 25μL of magnetic microbeads, 100μL of acridinium - labeled anti - human IgG, and 90μL of sample diluent are added sequentially. The mixture was subjected to two 10-min incubation cycles at 37°C, after which the magnetic microbeads were washed using the instrument’s automated program. After addition of pre-trigger and trigger solutions, relative light units (RLU) were measured and antibody concentrations were calculated from a calibration curve. Results are reported as AU/mL and cutoff values are shown in **Supplementary Table 2**.

### 3.3 ELISA for anti-Dsg1, anti-Dsg3, anti-BP180, anti-BP230

The commercially available MESACUP ELISA kits (Medical & Biological Laboratories Co. Ltd; MBL, Nagoya, Japan) was used to detect four AIBD autoantibodies. The test reader was blinded to clinical information as well as IIFT-BIOCHIP and/or CLIA results. The antigens used in the reagents were the same as those for CLIA.

Referring to the instructions, blood samples are centrifuged at 3000 rpm for 10 minutes to separate serum. Reagents are equilibrated to room temperature (18~25°C), and the concentrated washing buffer is diluted 1:10 with distilled water (1 part concentrated buffer + 9 parts distilled water). Patient samples are diluted 1:101 with sample diluent (e.g., 10 μL serum + 1.0 mL sample diluent; avoid pipetting for mixing). Add 100 μL of diluted samples, positive control, negative control, and standards to microplate wells and incubate at room temperature for 30 minutes. Discard liquid and wash 3 times with 300μL diluted washing buffer, allowing it to sit for 30~60 seconds before discarding. Invert and tap the plate on absorbent paper to remove residual buffer. Add 100 μL enzyme-conjugated anti-human IgG to each well, incubate at room temperature for 30 minutes, and repeat the washing step. Add 100 μL TMB (3, 3′,5,5′-Tetramethylbenzidine)/H_2_O_2_ substrate solution to wells and incubate in the dark for 15 minutes. Terminate the reaction with 100 μL 0.5M H_2_SO_4_ stop solution. Measure absorbance at 450 nm (reference: 620~650 nm) and calculate antibody concentration using a standard curve. Results are interpreted using pre-specified reagent cutoff values, as detailed in **Supplementary Table 2**.

### 3.4 IIFT-BIOCHIP for anti-Dsg1, anti-Dsg3, anti-BP180, anti-BP230

Antibody titers against Dsg1, Dsg3, BP180, and BP230 were measured through the IIFT-BIOCHIP: Dermatology Mosaic 60 kit (EUROIMMUN, Germany). The test reader was blinded to clinical information as well as ELISA and/or CLIA results. This kit combines a range of substrates (salt split skin and monkey oesophagus) with recombinant antigens, including transfected cells expressing Dsg1 and Dsg3, recombinant BP180-NC16A-4X, and recombinant BP230gC for the detection of antibodies targeting these antigens.

The test was performed according to the manufacturer’s instructions. Blood samples were centrifuged at 3000 rpm for 10 minutes to isolate serum. Reagents were equilibrated to room temperature (18~25°C), and FITC-labeled anti-human IgG was thoroughly mixed before use. Samples were diluted 1:10 in PBS-Tween buffer, with serial dilutions (1:10, 1:100, 1:1000, etc.) prepared for quantitative analysis. A 25 μL aliquot of diluted sample was added to the slide’s reaction area and incubated at room temperature for 30 minutes. The slide was washed with PBS-Tween buffer and immersed in a wash cup containing the same buffer for ≥5 minutes. Next, 25μL of FITC-labeled anti-human IgG was added to the reaction area and incubated at room temperature for 30 minutes in the dark, followed by repeating the washing step. For mounting, 10 μL of mounting medium was applied to a coverslip, and the slide was placed biological thin-film side down onto the coverslip. Observations were conducted using a fluorescence microscope (excitation: 488 nm, dichroic mirror: 510 nm, barrier filter: 520 nm), with a 20x objective for tissue analysis and 40x objective for cell-matrix evaluation. Results were interpreted using pre-specified reagent cutoff values, as detailed in **Supplementary Table 2**.

### 3.5 Assessment of disease severity

Disease severity was assessed using Body Surface Area (BSA) scoring, evaluated by an experienced clinician using the “rule of nines” (17) based on the distribution of blisters, erythema, and rash in AIBD patients. Briefly, the head and face each account for 9%, the neck for 1%, the torso for 18%, each arm for 9%, the genital area for 1%, and each leg for 18%. The total BSA is the sum of all affected areas. BSA defines disease severity as mild (<10%), moderate (10-50%), or severe (>50%).

### 3.6 Statistical analysis

Missing data for CLIA or IIFT-BIOCHIP and ELISA were excluded. Indeterminate results from CLIA or IIFT-BIOCHIP or ELISA were carefully reviewed and the final classification was based on the consensus interpretation. Statistical analyses were performed utilizing R Software (version 3.3.3), and GraphPad Prism 7.0. To evaluate agreement and identify potential biases, methods were compared through Pearson correlation analysis and Bland-Altman plots. Analysis of Variance (ANOVA) or non-parametric tests such as the Mann-Whitney U test was used to assess differences between groups. ROC and Area Under the Curve (AUC) values were used to determine diagnostic accuracy, sensitivity, and specificity, with statistical significance established at *P* < 0.05.

## 4 Results

### 4.1 Baseline demographics

The study included three groups (Healthy, Other skin diseases and AIBDs group, see flow chart in **Figure 1**), and baseline characteristics are shown in **Table 1**. Laboratory parameters and clinical characteristics of the subjects were collected (**Table 1**). Compared with Healthy and Other skin diseases group, AIBDs group had higher levels of whith blood cell (WBC), Neutrophil (NE), UREA and Urea/Creatinine (UREA/Cr, *p* < 0.001), and lower levels of Eosinophil (Eos, *p* < 0.001) and Hemoglobin (Hb, *p* = 0.002). Pemphigus patients had higher Hb levels (*p* < 0.05) and lower NE (*p* < 0.05) and Eos levels (*p* < 0.001) than BP patients (**Table 2**). Among AIBD patients, 126 cases (80.3%) were newly diagnosed. The clinical features of AIBD patients included blister (114 cases, 71.2%), skin rash (98 cases, 61.3%), pruritus (87 cases, 54.4%), mucosal lesions (19 cases, 11.9%) and Nikolsky sign (7 cases, 4.4%). According to the Body Surface Area (BSA) score, AIBD patients included 15 (11.0%) of mild, 22 cases (16.2%) of moderate, and 99 cases (72.8%) of severe cases (**Table 1**).

**Table 1:** Baseline characteristics of subjects.

Project	AIBDs	Healthy	Other skin diseases	Statistic	P
n	160	121	153		
sex, n (%)				2.985	0.225
male	86 (54)	52 (43)	83 (54)		
female	74 (46)	69 (57)	70 (46)		
age, Median (Q1,Q3)	67 (58.5, 74)	61 (54, 68)	58 (46, 66)	31.286	**< 0.001**
Groups					
	Pemphigus (%) = 54 (33.8)		Eczema (%) = 52(34.0)		
	Bullous Pemphigoid (%) = 106 (66.2)		Herpes zoster (%) = 50(32.7)		
			Psoriasis (%) = 51(33.3)		
First diagnosis = YES (%)	126 (80.3)				
Skin rash = YES (%)	98 (61.3)				
Blister = YES (%)	114 (71.2)				
Pruritus = YES (%)	87 (54.4)				
Mucosal lesion = YES (%)	19 (11.9)				
Nikolsky sign = YES (%)	7 (4.4)				
Area of lesion (mean (SD))	66.34 (30.53)				
Severity of lesion (%)					
Mild	15 (11.0)				
Moderate	22 (16.2)				
Severe	99 (72.8)				
WBC, Median (Q1,Q3)	8.3 (6.88, 10.95)	5.74 (5.06, 6.6)	6.36 (5.23, 7.88)	92.748	**< 0.001**
RBC, Median (Q1,Q3)	4.51 (4.04, 4.86)	3.18 (2.78, 3.85)	4.65 (4.17, 4.86)	116.31	**< 0.001**
NE, Median (Q1,Q3)	5.52 (4.21, 7.59)	0.1 (0.07, 0.15)	3.5 (2.75, 4.93)	246.49	**< 0.001**
Eos, Median (Q1,Q3)	0.12 (0.03, 0.37)	4.72 (4.48, 5.03)	0.15 (0.07, 0.28)	214.663	**< 0.001**
Hb, Median (Q1,Q3)	138 (125, 150.25)	144 (136, 153)	139 (126, 150)	12.796	**0.002**
Cr, Median (Q1,Q3)	65.1 (54.62, 79)	62.8 (54.95, 73.05)	66.4 (56.95, 75.5)	2.303	0.316
UREA, Median (Q1,Q3)	6.34 (4.73, 8.58)	5.21 (4.66, 5.92)	4.92 (3.69, 6.25)	28.697	**< 0.001**
UREA/Cr, Median (Q1,Q3)	91.64 (71.01, 122.71)	82.85 (72.9, 98.54)	62.95 (48.1, 95.25)	41.413	**< 0.001**
ALT, Median (Q1,Q3)	19.6 (12.35, 27.15)	20 (15.35, 26.65)	19.4 (12.1, 27.8)	0.634	0.728
AST, Median (Q1,Q3)	18.85 (14.4, 23.8)	20.9 (17.6, 24.05)	18.5 (15.2, 24.7)	4.837	0.089
AST/ALT, Median (Q1,Q3)	1.04 (0.75, 1.28)	1.02 (0.88, 1.23)	1.04 (0.83, 1.28)	1.151	0.562
CLIA					
Dsg1, Median (Q1,Q3)	1.43 (1.07, 17.47)	1.31 (1.25, 1.38)	1.32 (1.21, 1.61)	10.047	**0.007**
Dsg3, Median (Q1,Q3)	1.58 (1, 8.66)	1 (1, 1)	1 (1, 1.56)	75.854	**< 0.001**
Bp180, Median (Q1,Q3)	42.51 (1.92, 562.78)	1.73 (1.49, 2.1)	1.81 (1.46, 2.26)	92.508	**< 0.001**
Bp230, Median (Q1,Q3)	2.67 (1.67, 17.84)	1.98 (1.59, 2.67)	1.81 (1.32, 2.48)	32.164	**< 0.001**
IIFT-BIOCHIP					
Dsg1 = Positive(%)	41 (25.6)				
Dsg3 = Positive (%)	39 (24.4)				
BP180 (%)					
Weakly positive	6 (3.8)				
Positive	76 (47.5)				
Negative	78 (48.8)				
BP230 = Positive (%)	121 (75.6)				
ELISA					
Dsg1, Median (Q1,Q3)	8.86 (4.43, 20.47)				
Dsg3, Median (Q1,Q3)	4.28 (1.68, 9.58)				
Bp180, Median (Q1,Q3)	71.91 (16.37, 124.02)				
Bp230, Median (Q1,Q3)	20.87 (5.08, 104.44)				

Black bolded font indicates a significant difference between the three groups.

**Table 2:** Baseline characteristics of AIBD patients.

Project	Bullous pemphigoid (n = 106)	Pemphigus (n = 54)	Statistic	P
sex, n (%)			1.397	0.237
man	61 (58)	25 (46)		
woman	45 (42)	29 (54)		
age, Median (Q1,Q3)	70 (63, 76)	58 (49, 66.75)	4475.5	< 0.001
WBC, Median (Q1,Q3)	8.63 (7.1, 11.14)	7.68 (6.43, 9.6)	2547	0.075
RBC, Median (Q1,Q3)	4.45 (3.92, 4.85)	4.64 (4.18, 4.9)	1725.5	0.067
NE, Median (Q1,Q3)	5.91 (4.55, 7.87)	4.61 (3.87, 7.11)	2604.5	0.042
Eos, Median (Q1,Q3)	0.16 (0.04, 0.62)	0.06 (0.01, 0.16)	2916	< 0.001
Hb, Mean ± SD	134.54 ± 19	140.9 ± 16	-2.051	0.043
Cr, Median (Q1,Q3)	66.2 (55, 79.4)	62.2 (54, 79)	1631.5	0.745
UREA, Median (Q1,Q3)	6.4 (4.87, 8.87)	5.77 (4.47, 7.55)	1815.5	0.177
ALT, Median (Q1,Q3)	19.8 (12.2, 31.3)	19.4 (14, 24.3)	1637.5	0.881
AST, Median (Q1,Q3)	18.6 (13.9, 23.95)	19.1 (15.9, 23.5)	1573	0.844
Dsg1, Median (Q1,Q3)	1.26 (1, 1.47)	132.15 (15.62, 671.11)	277.5	< 0.001
Dsg3, Median (Q1,Q3)	1 (1, 2.19)	192.52 (2.22, 673.12)	916	< 0.001
Bp180, Median (Q1,Q3)	451.61 (34.64, 706.86)	1.85 (1.51, 2.33)	5020.5	< 0.001
Bp230, Median (Q1,Q3)	3.85 (2.1, 92.12)	1.95 (1.38, 2.67)	4220	< 0.001
First diagnosis, n (%)			0.125	0.724
NO	19 (18)	12 (22)		
YES	84 (82)	42 (78)		
Skin rash, n (%)			0.781	0.377
NO	38 (36)	24 (44)		
YES	68 (64)	30 (56)		
Blister, n (%)			0	1
NO	30 (28)	16 (30)		
YES	76 (72)	38 (70)		
Pruritus, n (%)			24.889	< 0.001
NO	33 (31)	40 (74)		
YES	73 (69)	14 (26)		
Mucosal lesion, n (%)			22.059	< 0.001
NO	103 (97)	38 (70)		
YES	3 (3)	16 (30)		
Nikolsky sign, n (%)			Fisher	< 0.001
NO	106 (100)	47 (87)		
YES	0 (0)	7 (13)		
Area of lesion, Median (Q1,Q3)	80 (64, 90)	76 (9, 90)	2555.5	0.017
Severity of lesion, n (%)			Fisher	< 0.001
Mild	2 (2)	13 (29)		
Moderate	13 (14)	9 (20)		
Severe	76 (84)	23 (51)		

### 4.2 Performance evaluation results of CLIA

This part of the study was carried out in strict accordance with the industry standard documents. The results of the precision experiments showed that the four autoantibody detection reagents met the requirements with a repeatability coefficient of variation (CV) ≤ 10.0% and a reproducibility coefficient of variation (CV) ≤ 15.0% (**Table 3**).

**Table 3:** Evaluation of the precision of CLIA.

Project	Sample	Mean (AU/mL)	Repeatability		Between-Run		Between-Day		Between-Site		Reproducibility	
			SD	CV(%)	SD	CV(%)	SD	CV(%)	SD	CV(%)	SD	CV(%)
Dsg1	Serum 1	10.45	0.406	3.89%	0.091	0.87%	0.106	1.01%	0.487	4.66%	0.649	6.21%
	Serum 2	22.42	0.853	3.80%	0.105	0.47%	0.185	0.83%	0.712	3.18%	1.132	5.05%
	Serum 3	331.86	9.477	2.86%	3.966	1.20%	1.294	0.39%	10.638	3.21%	14.845	4.47%
Dsg3	Serum 1	6.16	0.16	2.60%	0.06	0.97%	0.05	0.81%	0.28	4.55%	0.33	5.36%
	Serum 2	22.86	0.67	2.91%	0.11	0.50%	0.09	0.38%	0.88	3.85%	1.11	4.87%
	Serum 3	210.95	6.16	2.92%	0.69	0.33%	1.87	0.89%	8.38	3.97%	10.59	5.02%
BP180	Serum 1	5.00	0.154	3.08%	0.041	0.82%	0.018	0.36%	0.218	4.36%	0.271	5.42%
	Serum 2	23.06	0.695	3.01%	0.109	0.47%	0.109	0.47%	0.820	3.56%	1.086	4.71%
	Serum 3	79.83	1.926	2.41%	0.523	0.66%	0.187	0.23%	2.685	3.36%	3.351	4.20%
BP230	Serum 1	11.48	0.260	2.26%	0.095	0.83%	0.044	0.38%	0.397	3.46%	0.486	4.23%
	Serum 2	23.53	0.597	2.54%	0.247	1.05%	0.122	0.52%	0.848	3.60%	1.073	4.56%
	Serum 3	203.97	4.260	2.09%	0.557	0.27%	1.697	0.83%	6.261	3.07%	7.781	3.81%

**Figure 2:**
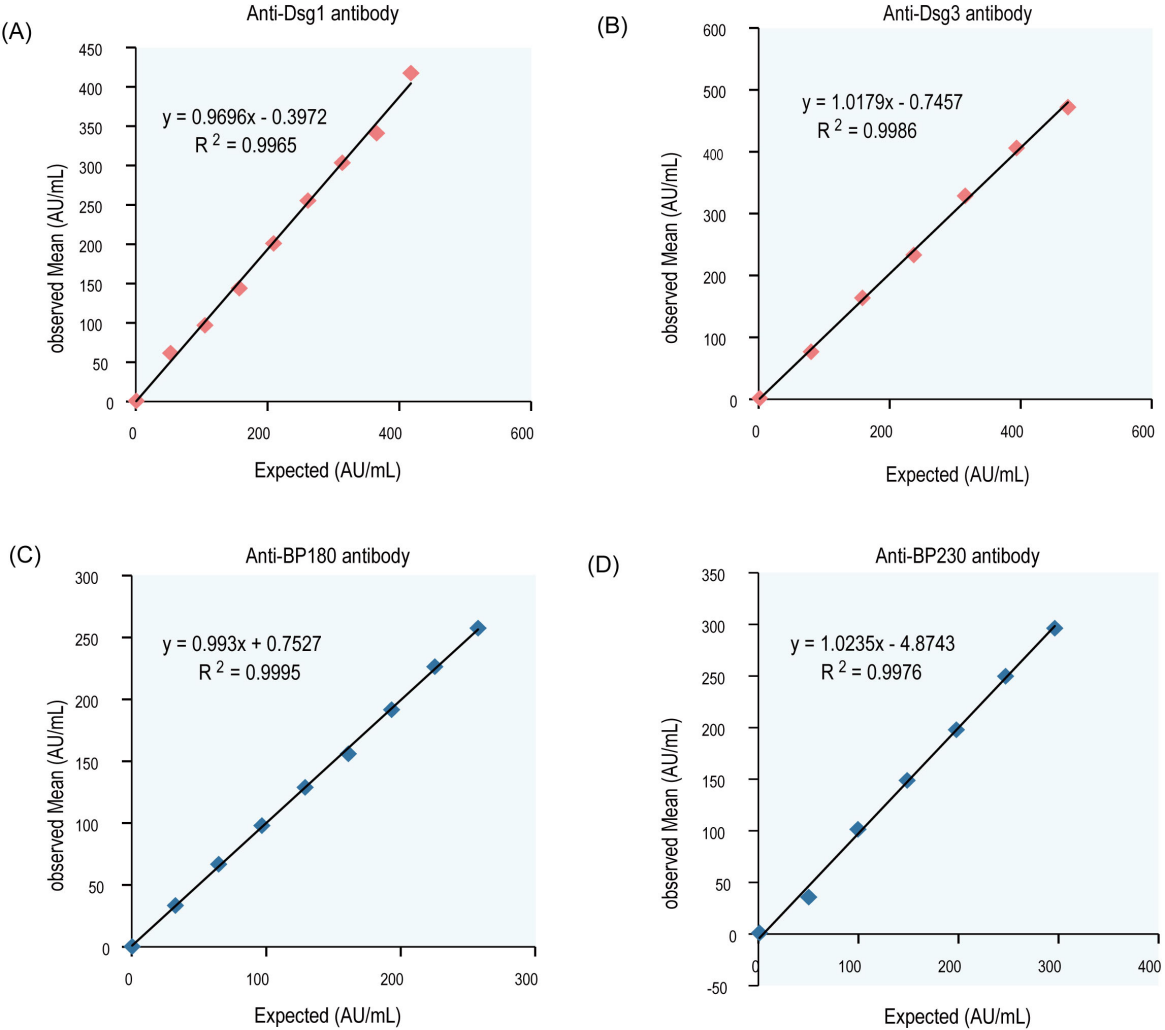
Linear curve of antibodies (Dsg1, Dsg3, Bp180, and Bp230). Mean results of the observed values were plotted against the target value, the correlation coefficient r≥0.99. **(A)** Dsg1, **(B)** Dsg3, **(C)** BP180, **(D)** BP230.

Interference experiments were evaluated for hemoglobin, bilirubin, triglycerides, total serum protein, RF, HAMA, and ANA, which showed that their induced values were within ±10.0%, confirming that there was no significant interference (**Tables 4**, **5**). The four CLIA reagents LoB, LoD, and LoQ are shown in **Table 6**. LoB, LoD, and LoQ were 0.71 0.91 1.73 AU/mL for anti-Dsg1, 1.09 1.47 1.75 AU/mL for anti-Dsg3, 0.31 0.60 1.73 AU/mL for BP180, and 0.30 0.44 2.00 AU/mL for anti-BP230 antibodies, respectively. The linear ranges of anti-Dsg1, Dsg3, BP180, and BP230 antibodies were 1.73~400 AU/mL (R^2^ = 0.9965), 1.75~400 AU/mL (R^2^ = 0.9986), 1.73~480 AU/mL (R^2^ = 0.9995), and 2.00 ~ 250AU/mL (R^2^ = 0.9976), respectively (**Table 7**; **Figure 2**).

**Table 4:** Evaluation of the anti-interference ability of CLIA.

Inteferon (conc.)	Dsg1-dobs		Dsg3-dobs		BP180-dobs		BP230-dobs	
	Negative sample	Weakly positive samples	Negative sample	Weakly positive samples	Negative sample	Weakly positive samples	Negative sample	Weakly positive samples
Hemoglobin	-3.75%	-1.86%	-0.30%	-5.32%	-3.59%	-0.98%	2.20%	1.55%
Bilirubin	2.05%	1.20%	-0.12%	-5.20%	-1.40%	-2.10%	3.72%	4.93%
Triglyceride	-1.52%	-2.93%	-0.10%	-0.66%	1.19%	1.23%	-3.14%	-3.12%
Total serum protein	-0.73%	-0.17%	-0.44%	-7.42%	-1.99%	-1.17%	3.15%	1.21%
RF	-0.35%	-1.38%	-0.25%	-4.87%	-3.94%	-0.26%	-1.87%	-1.05%
HAMA	-0.12%	-0.86%	-0.05%	-5.95%	4.71%	2.67%	-0.67%	-1.15%
ANA	-0.12%	-2.75%	-0.26%	-3.78%	1.00%	1.16%	-2.39%	-2.26%

**Table 5:** Tolerance limits of interference substances for CLIA.

Project	Hemoglobin (mg/dL)	Bilirubin (mg/dL)	Triglycerides (mg/dL)	Total serum protein (g/dL)	RF (IU/mL)	HAMA (ng/mL)	ANA (AU/mL)
Dsg1	≤1000	≤40	≤2000	≤10	≤2000	≤600	≤500
Dsg3	≤1000	≤40	≤2000	≤6	≤1000	≤600	≤500
BP180	≤1000	≤40	≤2000	≤10	≤1000	≤600	≤500
BP230	≤1000	≤40	≤2000	≤10	≤1000	≤600	≤500

**Table 6:** Performance characteristics of iFlash assay.

Project	LoB (AU/mL)	LoD (AU/mL)	LoQ (AU/mL)
Dsg1	0.71	0.91	1.73
Dsg3	1.09	1.47	1.75
BP180	0.31	0.60	1.73
BP230	0.30	0.44	2.00

**Table 7:** Evaluation of the linear range of the iFlash assay.

Project	Lower limit (AU/mL)	Upper limit (AU/mL)
Dsg1	1.73	400
Dsg3	1.75	400
BP180	1.73	480
BP230	2.00	250

**Figure 3:**
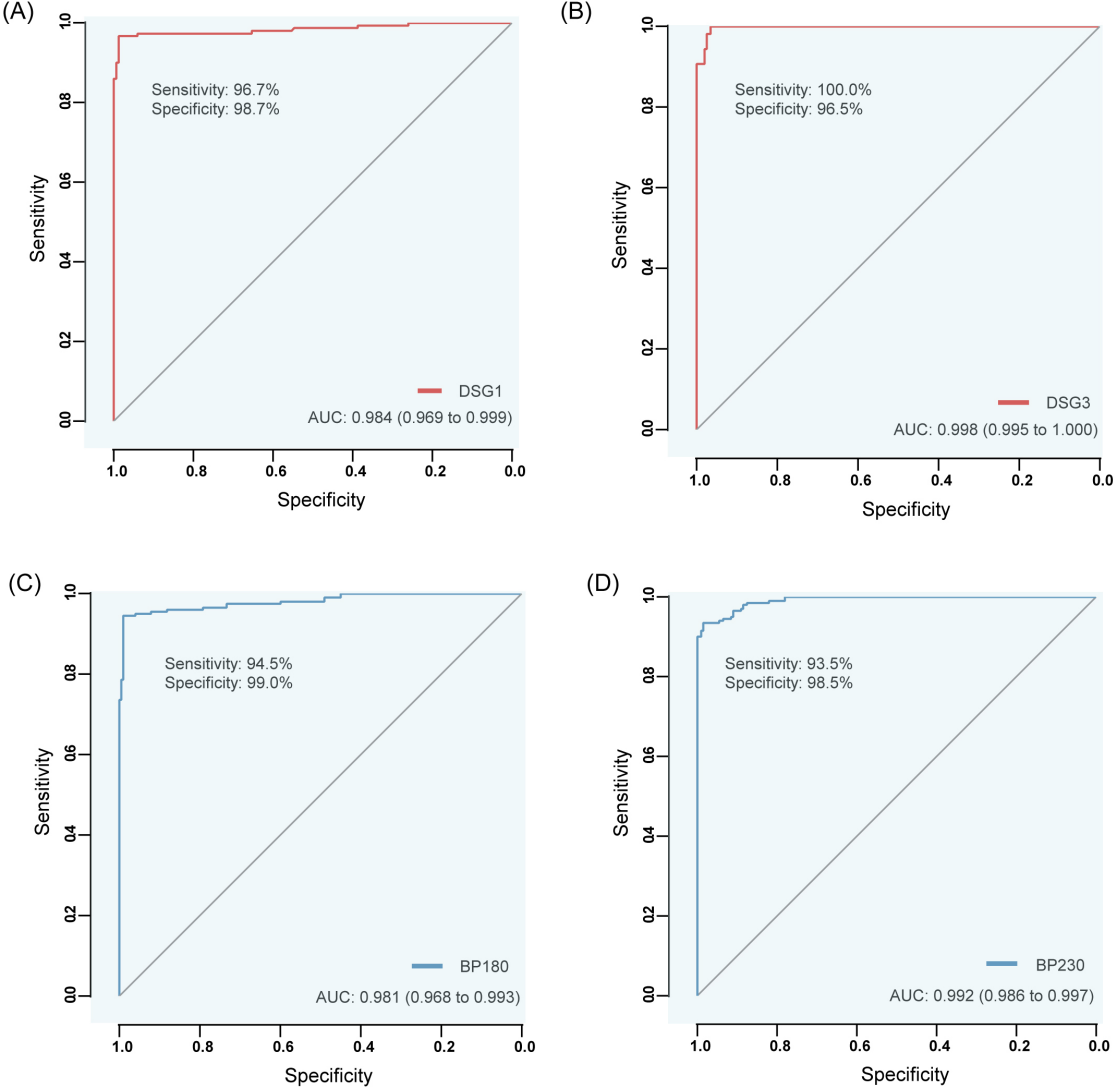
ROC curves of anti-Dsg1, Dsg3, BP180 and BP230 antibodies by CLIA differentiating between diseased (confirmed positive by similar commercially available products) and healthy populations. **(A)** Dsg1, **(B)** Dsg3, **(C)** BP180, **(D)** BP230.

The cut off values (positive assay values) for anti-Dsg1, Dsg3, BP180, and BP230 antibodies were determined and validated for patient samples (confirmed positive by marketed products of the same type) and for samples from healthy populations. The AUC values of anti-Dsg1, Dsg3, BP180, and BP230 antibodies were 0.984 (sensitivity 96.7%, specificity 98.7%, 95%CI: 0.969~0.999), 0.998 (sensitivity 100.0%, specificity 96.5%, 95%CI: 0.995~1.000), 0.981 (sensitivity 94.5%, specificity 99.0%, 95%CI: 0.968~0.993), and 0.992 (sensitivity 93.5%, specificity 98.5%, 95%CI: 0.986~0.997) for the diagnosed patients and the healthy population, respectively, and the critical luminescence value (RLU) concentration was set to 20 AU/mL based on the results of the Yoden index as the cut off value for the four autoantibody detection reagents (**Table 8**; **Figure 3**).

**Table 8:** Evaluation of cutoff values for the CLIA.

Project		Clinical status		Total	Sensitivity	Specificity	Kappa
		Disease	Health				
Dsg1							
Test results	Positive	48	0	48	96%	100%	0.96
	Negative	2	50	52			
Total		50	50	100			
Dsg3							
Test results	Positive	20	0	20	100%	100%	1
	Negative	0	20	20			
Total		20	20	40			
BP180							
Test results	Positive	47	1	48	94%	98%	0.92
	Negative	3	49	52			
Total		50	50	100			
BP230							
Test results	Positive	47	1	48	94%	98%	0.92
	Negative	3	49	52			
Total		50	50	100			

### 4.3 Distribution of antibodies specific to Dsg1, Dsg3, BP180 and BP230 using CLIA

**Figure 4:**
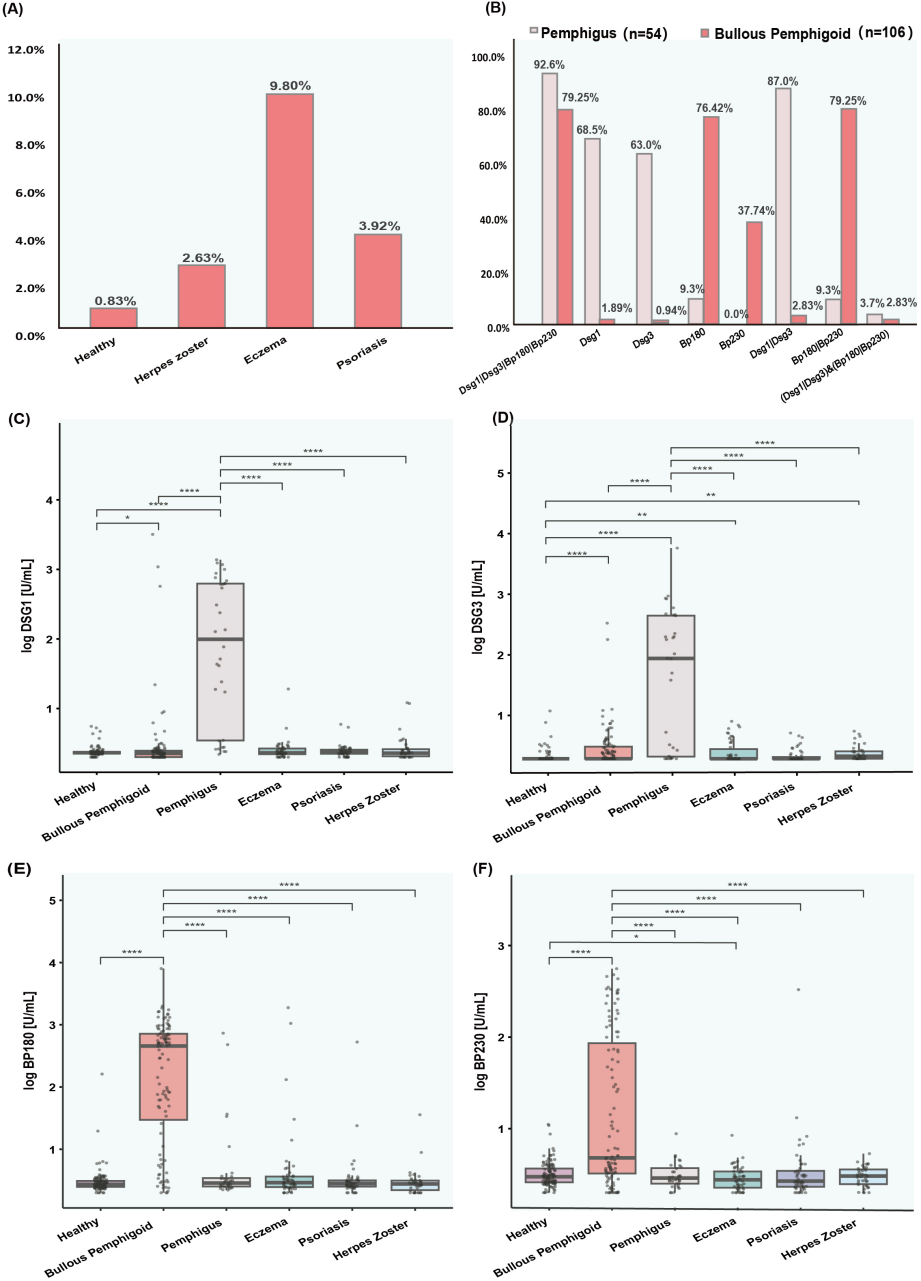
Prevalence and levels of antibodies (Dsg1, Dsg3, Bp180, and Bp230) in various patient groups. **(A)** Bar chart showing the percentage of positive results for autoantibodies in healthy individuals and populations with diseases easily confused by AIBD. **(B)** Bar chart displaying the prevalence of AIBD autoantibodies in patients with pemphigus and bullous pemphigoid. **(C–F)** Box plots showing the levels of autoantibodies (Dsg1, Dsg3, Bp180, and Bp230) in healthy controls and various patient groups. The y axis represents the log transformed antibody levels in arbitrary units per milliliter AU/ml. **(C)** Dsg1 levels. **(D)** Dsg3 levels. **(E)** Bp180 levels. **(F)** Bp230 levels.

92.6% of pemphigus patients and 79.25% of BP patients were positive for at least one AIBD autoantibody (Dsg1, Dsg3, BP180, BP230). Among them, 87% of patients with pemphigus were positive for either anti-Dsg1 and anti-Dsg3 antibodies, and 79.25% of patients with BP were positive for either anti-BP180 and anti-BP230 antibodies (**Figure 4B**). The positive rates of anti-Dsg1, Dsg3, BP180 and BP230 antibodies were 68.5%, 63.0%, 9.3% and 0% in patients with pemphigus, and 1.89%, 0.94%, 76.42% and 37.74% in patients with BP, respectively. The levels of anti-Dsg1 and anti-Dsg3 were significantly higher in pemphigus patients, whereas the levels of anti-BP180 and anti-BP230 were significantly elevated in BP patients compared to both disease control patients and healthy volunteers (**Figures 4C–F**). Analyzing the subtypes of pemphigus, Dsg3 levels were significantly higher in patients with PV than in patients with PF, and Dsg1 antibodies also differed between the two, but not as significantly as Dsg3 (**Supplementary Figure 1**).

The reference intervals for antibodies (Dsg1, Dsg3, BP180, and BP230) in healthy people and in patients with diseases easily confused by AIBD were determined by distributions of 2.5 and 97.5%, as shown in **Supplementary Table 3**. In addition, 9.80%, 3.92%, 2.63% of eczema, psoriasis, and herpes zoster patients were positive for at least one AIBD autoantibody, respectively (**Figure 4A**). Anti-Dsg3 and anti-BP230 antibody levels were significantly higher in eczema patients, while anti-Dsg3 antibody levels were also significantly higher in herpes zoster patients than in the healthy population (**Figures 4C–F**).

### 4.4 Consistency comparison of different detection methods

**Figure 5:**
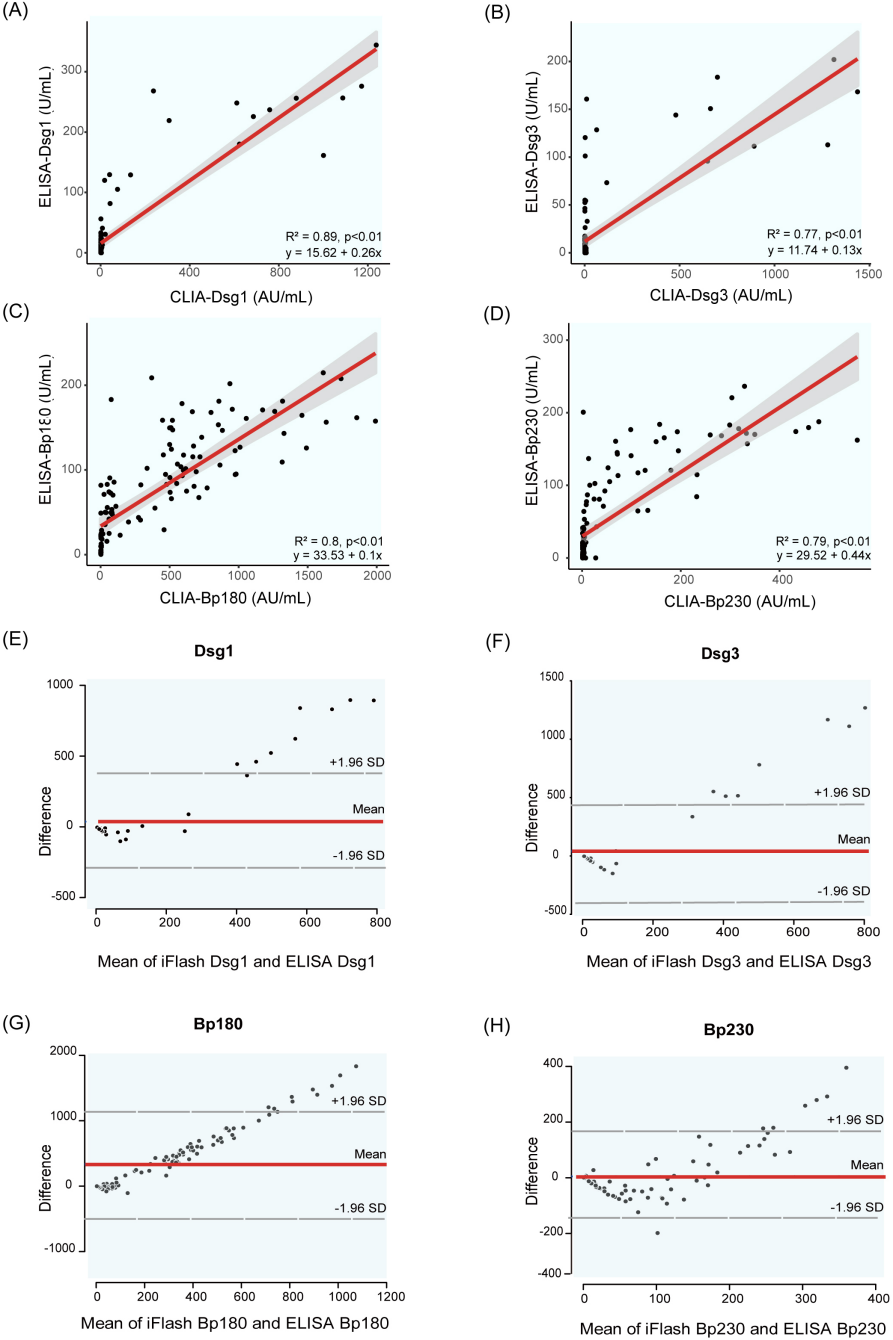
Correlation and Bland Altman analysis of Dsg1, Dsg3, BP180 and BP230 autoantibody levels measured by CLIA and ELISA. **(A–D)** show scatter plots illustrating the correlation between autoantibody levels measured by the two methods: **(A)** Dsg1, **(B)** Dsg3, **(C)** BP180, **(D)** BP230. **(E–H)** present Bland-Altman plots, which depict the differences between the two methods plotted against their averages. The dashed lines represent the mean difference and the limits of agreement (mean ± 1.96 standard deviations). These plots assess the agreement between the two methods for measuring Dsg1 **(E)**, Dsg3 **(F)**, BP180 **(G)**, and BP230 **(H)** antibody levels.

To assess the performance consistency between the CLIA and ELISA methods, 126 clinical samples were analyzed. Pearson’s correlation analysis showed strong correlations between CLIA and ELISA results of AIBD antibodies detection (R² =0.77 ~ 0.89, *p* < 0.01) (**Figures 5A–D**), and these findings indicated good concordance between the two assays. Additionally, the scatter distribution in the Bland Altman plots revealed that most of the data points were clustered around the mean line, suggesting that the differences between the two methods were still within acceptable range (**Figures 5E–H**). However, as the concentration of antibodies detected by CLIA increased, the scatter points deviated progressively upward from the mean line, suggesting that CLIA has a wider detection range and higher sensitivity than ELISA, especially when detecting high concentrations of antibody.

To evaluate the consistency between the CLIA, IIFT-BIOCHIP, and ELISA methods, a confusion matrix was constructed, and positive percent agreement (PPA) and negative percent agreement (NPA) were calculated for each pair of methods across the detection of anti-Dsg1, anti-Dsg3, anti-BP180, and anti-BP230 autoantibodies (**Table 9**). The CLIA method showed strong agreement with IIFT-BIOCHIP, with PPAs of 90%, 90%, 99%, and 92% for anti-Dsg1, anti-Dsg3, anti-BP180, and anti-BP230, respectively, and NPAs of 98%, 100%, 94%, and 97%, leading to consistency values of 96%, 98%, 96%, and 96%, respectively. In comparison, CLIA and ELISA showed lower PPAs for all antibodies, with values of 50%, 50%, 85%, and 49% for anti-Dsg1, anti-Dsg3, anti-BP180, and anti-BP230, respectively, though both assays exhibited high NPA, with 100% NPA for anti-Dsg1, anti-Dsg3, and anti-BP180, and 98% for anti-BP230. The comparison between IIFT-BIOCHIP and ELISA revealed that while both methods showed 100% NPA for all four antibodies, the PPA for ELISA was notably lower, with values of 50%, 50%, 82%, and 49% for anti-Dsg1, anti-Dsg3, anti-BP180, and anti-BP230, respectively. The consistency values for these antibodies were 87%, 92%, 86%, and 68%, respectively (**Supplementary Table 5**). These results suggest that while the CLIA and IIFT-BIOCHIP methods demonstrate strong agreement, ELISA showed reduced PPA.

**Table 9:** Consistency comparison of different methods for the detection of AIBD autoantibodies.

Dsg1	Parameter	IIFT-BIOCHIP	ELISA
CLIA	PPA	90%	50%
	NPA	98%	100%
	Consistency values	96%	87%
Dsg3		IIFT-BIOCHIP	ELISA
CLIA	PPA	90%	50%
	NPA	100%	100%
	Consistency values	98%	92%
BP180		IIFT-BIOCHIP	ELISA
CLIA	PPA	99%	85%
	NPA	94%	100%
	Consistency values	96%	88%
BP230		IIFT-BIOCHIP	ELISA
CLIA	PPA	92%	49%
	NPA	97%	98%
	Consistency values	96%	67%
Dsg1 or Dsg3		IIFT-BIOCHIP	ELISA
CLIA	PPA	96%	43%
	NPA	99%	100%
	Consistency values	98%	81%
BP180 or BP230		IIFT-BIOCHIP	ELISA
CLIA	PPA	100%	88%
	NPA	88%	100%
	Consistency values	94%	90%

PPA, positive percent agreements; NPA, negative percent agreements

Analyzing AIBD patients with anti-Dsg1 or anti-Dsg3 antibody positivity and anti-BP180 or anti-BP230 antibody positivity, CLIA and IIFT-BIOCHIP had high consistency (98% and 94% respectively), with PPA of 96% and 100%, NPA of 99% and 88%. However, the consistency between CLIA and ELISA was poorer (81% and 90% respectively) with low PPA of 43% and 88%, despite both having NPA of 100% (**Table 9**). The concordance of the three assays in AIBD subtypes is shown in **Supplementary Table 6**. CLIA showed better concordance with IIFT-BIOCHIP in BP patients and with ELISA in pemphigus patients.

In summary, both the CLIA and IIFT-BIOCHIP methods demonstrated very good agreement across all indicators (anti-Dsg1, Dsg3, Bp180, and BP230 antibodies), while the CLIA and ELISA methods exhibited relatively good agreement, their comparatively lower consistency in PPA rates was observed.

### 4.5 Evaluation of CLIA in the diagnosis of AIBD and its subtypes

**Figure 6:**
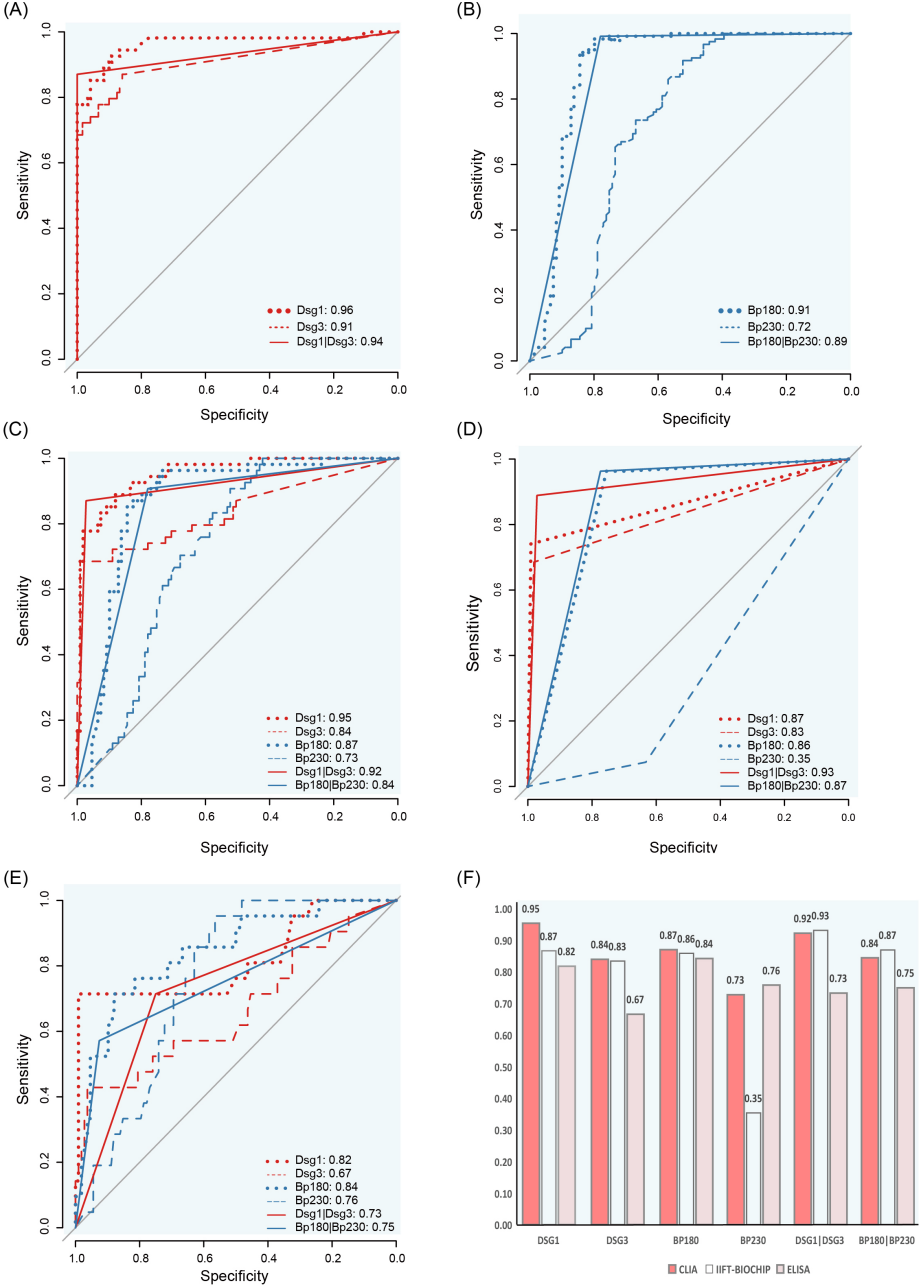
ROC curves and AUC comparison for CLIA, IIFT-BIOCHIP and ELISA antibody levels. **(A)** ROC curves for distinguishing pemphigus from healthy controls using CLIA. **(B)** ROC curves for distinguishing bullous pemphigoid from healthy controls using CLIA. C-E: ROC curves for differentiating pemphigus and bullous pemphigoid using CLIA **(C)**, IIFT-BIOCHIP **(D)** or ELISA **(E)**. **(F)** Bar chart comparing AUC values for CLIA, IIFT-BIOCHIP and ELISA.

The Receiver Operating Characteristic (ROC) curve was employed to evaluate the diagnositic value of CLIA in AIBDs. The results demonstrated that anti-Dsg1 and anti-Dsg3, exhibited superior performance in differentiating between healthy individuals and pemphigus, with AUC values of 0.96 (95%CI: 0.93~1) for anti-Dsg1 and 0.91 (95%CI:0.86~0.96) for anti-Dsg3 (**Figure 6A**). The AUC values in differentiating between healthy individuals and BP with anti-BP180 and anti-BP230 antibodies were 0.91 (95%CI: 0.86~95) and 0.72 (95%CI: 0.65~0.80), respectively (**Figure 6B**).

Notably, in the discrimination of pemphigus and BP, for Dsg1, Dsg3, and BP180 antibodies, the AUC values of CLIA were 0.95 (95%CI: 0.92~0.98), 0.84 (95%CI: 0.77~0.91), and 0.87 (95%CI: 0.82~0.93), which were the best among the three methods (**Figures 6D–F**). For BP230, ELISA showed the best differential diagnosis ability with an AUC value of 0.76 (95%CI: 0.67~0.85), while the AUC value of CLIA method was 0.73 (95%CI: 0.66~0.81). Combined detection of Dsg1 and Dsg3, BP180 and BP230, the CLIA method showed excellent performance similar to IIFT-BIOCHIP, both of which were superior to the ELISA method (**Figure 6F**).

### 4.6 Correlation of AIBD antibodies with clinical features and disease severity

Patients with BP had more frequent pruritus (69%), higher BSA scores and proportion of severe patients (84%), while patients with pemphigus had more mucosal lesions (30%) and Nikolsky sign symptoms (13%), and the proportion of severe skin lesions was 51% (**Table 2**). Patients with mucosal lesion and Nikolsky sign had higher levels of anti-Dsg1 and anti-Dsg3 antibodies, and patients with pruritus had higher levels of anti-BP180 and anti-BP230 antibodies (**Supplementary Table 4**). To further clarify the correlation between Dsg1 and Dsg3 and clinical symptoms, we divided patients with pemphigus into PV (Dsg1^-^Dsg3^+^, Dsg1^+^Dsg3^+^) and PF (Dsg1^+^Dsg3^-^). Compared with Dsg3-negative PF patients, Dsg3-positive PV patients (Dsg1^-^Dsg3^+^, Dsg1^+^Dsg3^+^) were more prone to mucosal lesion, reflecting the central role of Dsg3 antibodies in mucosal damage, in agreement with the clinical consensus (**Supplementary Figure 2**) (3, 18). In pemphigus patients, anti-Dsg1 antibody level was higher in the severe group than in the mild group. However, no correlation between BSA score and anti-Dsg3, BP180, and BP230 antibody levels was observed (**Supplementary Table 4**).

## 5 Discussion

We introduce a novel two-step, sandwich paramagnetic particles acridine ester CLIA for detecting autoantibodies to Dsg1, Dsg3, BP180, and BP230—key biomarkers for AIBDs. This fully automated method demonstrated strong agreement with IIFT-BIOCHIP and excellent diagnostic performance in distinguishing between pemphigus and BP patients, achieving AUC values of 0.92 (95%CI: 0.92~0.98) (anti-Dsg1 or anti-Dsg3 antibody) and 0.84 (95%CI: 0.77~0.91) (anti-BP180 or anti-BP230 antibody). It outperformed ELISA (AUC: 0.73 (95%CI: 0.62~0.84) and 0.75 (95%CI: 0.64~0.86)) and was comparable to IIFT-BIOCHIP technology (AUC: 0.93 (95%CI: 0.89~0.98) and 0.87 (95%CI: 0.82~0.92)). Additionally, our data suggest that the CLIA method offers a wider detection range and superior sensitivity compared to ELISA. Autoantibody levels correlated with disease severity and specific clinical symptoms, with elevated anti-Dsg3 associated with mucosal lesions, elevated anti-Dsg1/anti-Dsg3 associated with Nikolsky sign, and elevated anti-BP180/anti-BP230 levels linked to pruritus. These findings highlight the diagnostic and prognostic potential of this method for AIBDs.

Diagnosing AIBDs relies on clinical presentation, tissue-bound and circulating autoantibodies (19, 20). Rapid and accurate diagnosis is crucial, as pemphigus and BP often require immediate, aggressive treatment. Therefore, sensitive and specific antibody detection methods are essential.

Direct immunofluorescence (DIF), the gold standard for detecting tissue-bound autoantibodies, has high diagnostic accuracy (sensitivity: 76–98.1%, specificity: 99%) (21–23). However, it provides limited antigen information, making subtype differentiation difficult, and requires invasive biopsies, increasing the risk of errors and false positives (6, 22, 24).

IIFT-BIOCHIP is widely used to detect circulating autoantibodies. Traditional IIF uses tissue substrates like monkey esophagus and salt-split skin to identify autoantibodies, with sensitivity rates for pemphigus and BP ranging from 73.2% to 90% (25–27). While less invasive than DIF, traditional IIF is time-consuming, multi-step, and often requires multiple tests for accurate diagnosis. The IIFT-BIOCHIP used here improves upon traditional methods by incorporating tissue substrates with recombinant antigens (Dsg1, Dsg3, BP180, and BP230) into a one-step diagnostic approach. Our findings revealed good diagnostic value, with AUCs of 0.93 (95%CI: 0.89–0.98) for anti-Dsg1/anti-Dsg3 and 0.87 (95%CI: 0.82–0.92) for anti-BP180/anti-BP230 in differentiating pemphigus and BP, consistent with prior studies (2, 28). However, IIFT-BIOCHIP’s subjective nature may result in interpretation bias without adequate training (2).

ELISA assays are another widely used method for quantifying specific autoantibodies. In this study, ELISA demonstrated moderate diagnostic performance (AUC: 0.73–0.75). While ELISA is effective, its limited throughput, long incubation times, and equipment requirements hinder efficiency in clinical settings.

Unlike ELISA, which takes 2.5 hours, the CLIA method offers full automation, faster processing (35 minutes), and higher throughput. It simultaneously measures four autoantibodies—anti-Dsg1, anti-Dsg3, anti-BP180, and anti-BP230—from a single serum sample tube, enabling differentiation of AIBD subtypes. This makes the CLIA ideal for timely and efficient AIBD diagnosis.

The CLIA showed excellent diagnostic performance, achieving AUC values of 0.92 (95%CI: 0.87~0.97) for anti-Dsg1/anti-Dsg3 and 0.84 (95%CI: 0.79~0.90) for anti-BP180/anti-BP230. Correlation analyses revealed strong concordance between CLIA and ELISA results (R²: 0.77–0.89), though Bland-Altman plots indicated a wider detection range for CLIA. This broader range can reduce the need for sample dilution, addressing a key limitation of ELISA (29, 30).

Notably, CLIA showed higher agreement with IIFT-BIOCHIP (96–98%) but slightly lower agreement with ELISA (67–92%), particularly in positive agreement (49-85%). When examining discrepancies, 81.5% and 100% of samples positive for anti-Dsg1 and anti-Dsg3 antibodies, respectively, by ELISA but negative by CLIA were from BP patients, indicating potential false-positive results in ELISA (**Supplementary Table 7**). The CLIA method’s higher sensitivity and broader detection range enhance its ability to detect true differences in antibody titers, even at low antibody titers.

A similar chemiluminescent enzyme immunoassay (CLIEA) for diagnosing pemphigus and BP was evaluated, but it measured only three autoantibodies—anti-Dsg1, anti-Dsg3, and anti-BP180—excluded anti-BP230 (30). In contrast, the CLIA here includes anti-BP230, offering a more complete diagnostic profile. Additionally, unlike the CLIEA that uses alkaline phosphatase-labeled antibodies and a substrate, our CLIA uses acridine ester-labeled antibodies, eliminating the need for a substrate and making it faster to perform. These features make our CLIA more efficient and accurate for diagnosing AIBDs.

European guidelines recommend monitoring disease activity during treatment to guide appropriate therapeutic strategies (7). We demonstrated a correlation between anti-Dsg1 antibody titers and skin injury BSA scores, while anti-Dsg3, anti-BP180, and anti-BP230 titers did not show significant correlation (**Supplementary Table 4**). This aligns with previous studies showing that anti-Dsg1 titers correlated with skin severity (29), while anti-BP230 titer did not correlate with disease severity (31). Anti-Dsg3 is known to correlate with mucosal symptom severity, though its relationship with skin symptoms remains controversial (29, 32). Our findings on anti-BP180 contradict previous reports linking anti-BP180 titers with the Autoimmune Bullous Skin Disorder Intensity Score (ABSIS) (31), possibly due to the high proportion of severe BP cases in our cohort (84%) (**Table 2**).

ELISA reflects disease activity in later stages (29, 32, 33), but antibody changes often lag behind symptom remission in the initial treatment phase (3). IIFT-BIOCHIP titers are inconsistent and unreliable for monitoring severity (33–39). This discrepancy may result from the relatively small short-term changes in antibody titers, despite their clinical significance. CLIA, with its high sensitivity, holds promise for more accurate early-stage monitoring and longitudinal follow-up, which is currently being investigated.

Our findings also revealed a correlation between autoantibody levels and diagnostically relevant clinical characteristics of major AIBDs. Consistent with previous studies (6, 7, 20, 40), patients with BP were generally older and more likely to present with pruritus, with higher BSA scores, and a greater proportion of severe cases (**Table 2**). In contrast, pemphigus patients frequently exhibited mucosal lesions, Nikolsky sign, and a moderate proportion of severe skin involvement. Elevated anti-Dsg3 associated with mucosal lesions, anti-Dsg1/anti-Dsg3 associated with Nikolsky sign, while anti-BP180/anti-BP230 levels were linked to pruritus (**Supplementary Table 4**). While these findings are in line with previous report (18, 41), and reflect hallmark features of pemphigus and BP, this is particularly significant for nonbullous pemphigoid (NBP), which constituted 28% of BP cases and is prone to misdiagnosis due to atypical presentations like pruritus without blisters (42). The findings underscore the importance of autoantibody testing in diagnosing and managing atypical AIBD cases.

CLIA identified AIBD autoantibodies in patients with other dermatological conditions, such as eczema (9.80%), psoriasis (3.92%), and herpes zoster (2.63%) (**Figure 4A**). Recent studies have reported the detection of BP autoantibodies in elderly patients presenting with nonbullous, and pruritic disorders who did not yet meet the full diagnostic criteria for BP (41). This suggests that the presence of BP autoantibodies in conditions like eczema may represent a preclinical stage of BP (43) or might reflect a diagnostic overlap or could be false-positive results, which could complicate the diagnosis of AIBDs in these patients. Hence, further research is required to confirm these findings and to better understand their clinical implications.

Despite promising results, limitations include the single-centr design and cross-sectional nature of this study. Multicenter, longitudinal studies are necessary to validate the CLIA’s utility in diverse populations and its potential for dynamic disease monitoring.

## 6 Conclusion

In conclusion, the novel CLIA, the first to cover four major AIBD autoantibodies (anti-Dsg1, anti-Dsg3, anti-BP180, and anti-BP230) on a fully automated platform, provides a reliable and efficient alternative to IIFT-BIOCHIP and outperforms ELISA in diagnosing AIBDs. Its strong diagnostic performance, ability to assess disease severity, and clinical relevance make it a valuable tool for managing pemphigus and BP. Future research on dynamic autoantibody monitoring could further enhance its clinical utility.

## Data Availability

The original contributions presented in the study are included in the article/**Supplementary Material**. Further inquiries can be directed to the corresponding author.

## References

[1] Hammers CM Stanley JR . Mechanisms of disease: pemphigus and bullous pemphigoid. Annu Rev Pathol. (2016) 11:175–97. doi: 10.1146/annurev-pathol-012615-044313, PMID: 26907530 PMC5560122

[2] Yang A Xuan R Melbourne W Tran K Murrell DF . Validation of the BIOCHIP test for the diagnosis of bullous pemphigoid, pemphigus vulgaris and pemphigus foliaceus. J Eur Acad Dermatol Venereol. (2020) 34:153–60. doi: 10.1111/jdv.15770, PMID: 31260565

[3] Amagai M Tanikawa A Shimizu T Hashimoto T Ikeda S Kurosawa M . Japanese guidelines for the management of pemphigus. J Dermatol. (2014) 41:471–86. doi: 10.1111/1346-8138.12486, PMID: 24909210

[4] Ghodsi SZ Chams-Davatchi C Daneshpazhooh M Valikhani M Esmaili N . Quality of life and psychological status of patients with pemphigus vulgaris using Dermatology Life Quality Index and General Health Questionnaires. J Dermatol. (2012) 39:141–4. doi: 10.1111/j.1346-8138.2011.01382.x, PMID: 21967321

[5] Ruocco V Ruocco E Lo Schiavo A Brunetti G Guerrera LP Wolf R . Pemphigus: etiology, pathogenesis, and inducing or triggering factors: facts and controversies. Clin Dermatol. (2013) 31:374–81. doi: 10.1016/j.clindermatol.2013.01.004, PMID: 23806154

[6] Schmidt E Goebeler M Hertl M Sárdy M Sitaru C Eming R . S2k guideline for the diagnosis of pemphigus vulgaris/foliaceus and bullous pemphigoid. J Dtsch Dermatol Ges. (2015) 13:713–27. doi: 10.1111/ddg.12612, PMID: 26110729

[7] Borradori L Van Beek N Feliciani C Tedbirt B Antiga E Bergman R . Updated S2 K guidelines for the management of bullous pemphigoid initiated by the European Academy of Dermatology and Venereology (EADV). J Eur Acad Dermatol Venereol. (2022) 36:1689–704. doi: 10.1111/jdv.18220, PMID: 35766904

[8] Schmidt E Zillikens D . Pemphigoid diseases. Lancet. (2013) 381:320–32. doi: 10.1016/S0140-6736(12)61140-4, PMID: 23237497

[9] Daniel BS Murrell DF . Review of autoimmune blistering diseases: the Pemphigoid diseases. J Eur Acad Dermatol Venereol. (2019) 33:1685–94. doi: 10.1111/jdv.15679, PMID: 31087464

[10] Kershenovich R Hodak E Mimouni D . Diagnosis and classification of pemphigus and bullous pemphigoid. Autoimmun Rev. (2014) 13:477–81. doi: 10.1016/j.autrev.2014.01.011, PMID: 24424192

[11] Wang M Li F Wang X Wang R Chen T Zhao J . BIOCHIP mosaic for the diagnosis of autoimmune bullous diseases in Chinese patients. Eur J Dermatol. (2020) 30:338–44. doi: 10.1684/ejd.2020.3839, PMID: 32969793

[12] Van de Gaer O de Haes P Bossuyt X . Detection of circulating anti-skin antibodies by indirect immunofluorescence and by ELISA: a comparative systematic review and meta-analysis. Clin Chem Lab Med. (2020) 58:1623–33. doi: 10.1515/cclm-2019-1031, PMID: 32335537

[13] Mee JB . Diagnostic techniques in autoimmune blistering diseases. Br J BioMed Sci. (2023) 80:11809. doi: 10.3389/bjbs.2023.11809, PMID: 38074463 PMC10704243

[14] Gambino CM Agnello L Lo Sasso B Scazzone C Giglio RV Candore G . Comparative analysis of BIOCHIP mosaic-based indirect immunofluorescence with enzyme-linked immunosorbent assay for diagnosing myasthenia gravis. Diagnostics (Basel). (2021) 11:2098. doi: 10.3390/diagnostics11112098, PMID: 34829445 PMC8619605

[15] van Beek N Holtsche MM Atefi I Olbrich H Schmitz MJ Pruessmann J . State-of-the-art diagnosis of autoimmune blistering diseases. Front Immunol. (2024) 15:1363032. doi: 10.3389/fimmu.2024.1363032, PMID: 38903493 PMC11187241

[16] Natrajan A Sharpe D Costello J Jiang Q . Enhanced immunoassay sensitivity using chemiluminescent acridinium esters with increased light output. Analytical Biochem. (2010) 406:204–13. doi: 10.1016/j.ab.2010.07.025, PMID: 20670613

[17] Wallace AB . The exposure treatment of burns. Lancet. (1951) 1:501–4. doi: 10.1016/S0140-6736(51)91975-7, PMID: 14805109

[18] Rahbar Z Daneshpazhooh M Mirshams-Shahshahani M Esmaili N Heidari K Aghazadeh N . Pemphigus disease activity measurements: pemphigus disease area index, autoimmune bullous skin disorder intensity score, and pemphigus vulgaris activity score. JAMA Dermatol. (2014) 150:266–72. doi: 10.1001/jamadermatol.2013.8175, PMID: 24429657

[19] China Dermatologist Association, Treatment Group, Chinese Society of Dermatology, Dermatology Branch of China International Exchange and Promotive Association for Medical and Health Care, National Clinical Research Center for Dermatologic and Immunologic Diseases, Rare Skin Diseases Committee, China Alliance for Rare Diseases . Guidelines for diagnosis and treatment of pemphigus in China. Chin J Dermatol. (2024) 57:873–86. doi: 10.35541/cjd.20240222

[20] Witte M Zillikens D Schmidt E . Diagnosis of autoimmune blistering diseases. Front Med (Lausanne). (2018) 5:296. doi: 10.3389/fmed.2018.00296, PMID: 30450358 PMC6224342

[21] Mysorekar VV Sumathy TK Shyam Prasad AL . Role of direct immunofluorescence in dermatological disorders. Indian Dermatol Online J. (2015) 6:172–80. doi: 10.4103/2229-5178.156386, PMID: 26009711 PMC4439745

[22] Buch AC Kumar H Panicker N Misal S Sharma Y Gore CR . A cross-sectional study of direct immunofluorescence in the diagnosis of immunobullous dermatoses. Indian J Dermatol. (2014) 59:364–8. doi: 10.4103/0019-5154.135488, PMID: 25071256 PMC4103273

[23] Helander SD Rogers RS 3rd . The sensitivity and specificity of direct immunofluorescence testing in disorders of mucous membranes. J Am Acad Dermatol. (1994) 30:65–75. doi: 10.1016/S0190-9622(94)70010-9, PMID: 8277034

[24] Boraiy L Fontao L . Michel’s transport medium as an alternative to liquid nitrogen for PCR analysis of skin biopsy specimens. Dermatopathology (Basel). (2014) 1:70–4. doi: 10.1159/000368347, PMID: 27047924 PMC4772930

[25] Harman KE Gratian MJ Bhogal BS Challacombe SJ Black MM . The use of two substrates to improve the sensitivity of indirect immunofluorescence in the diagnosis of pemphigus. Br J Dermatol. (2000) 142:1135–9. doi: 10.1046/j.1365-2133.2000.03538.x, PMID: 10848736

[26] MaChado P Michalaki H Roche P Gaucherand M Thivolet J Nicolas JF . Serological diagnosis of bullous pemphigoid (BP): comparison of the sensitivity of indirect immunofluorescence on salt-split skin to immunoblotting. Br J Dermatol. (1992) 126:236–41. doi: 10.1111/j.1365-2133.1992.tb00651.x, PMID: 1554599

[27] Sárdy M Kostaki D Varga R Peris K Ruzicka T . Comparative study of direct and indirect immunofluorescence and of bullous pemphigoid 180 and 230 enzyme-linked immunosorbent assays for diagnosis of bullous pemphigoid. J Am Acad Dermatol. (2013) 69:748–53. doi: 10.1016/j.jaad.2013.07.009, PMID: 23969034

[28] van Beek N Rentzsch K Probst C Komorowski L Kasperkiewicz M Fechner K . Serological diagnosis of autoimmune bullous skin diseases: prospective comparison of the BIOCHIP mosaic-based indirect immunofluorescence technique with the conventional multi-step single test strategy. Orphanet J Rare Dis. (2012) 7:49. doi: 10.1186/1750-1172-7-49, PMID: 22876746 PMC3533694

[29] Daneshpazhooh M Chams-Davatchi C Khamesipour A Mansoori P Taheri A Firooz A . Desmoglein 1 and 3 enzyme-linked immunosorbent assay in Iranian patients with pemphigus vulgaris: correlation with phenotype, severity, and disease activity. J Eur Acad Dermatol Venereol. (2007) 21:1319–24. doi: 10.1111/j.1468-3083.2007.02254.x, PMID: 17958835

[30] Fujio Y Kojima K Hashiguchi M Wakui M Murata M Amagai M . Validation of chemiluminescent enzyme immunoassay in detection of autoantibodies in pemphigus and pemphigoid. J Dermatol Sci. (2017) 85:208–15. doi: 10.1016/j.jdermsci.2016.12.007, PMID: 28012821

[31] Chou PY Yu CL Wen CN Tu YK Chi CC . Bullous pemphigoid severity and levels of antibodies to BP180 and BP230: A systematic review and meta-analysis. JAMA Dermatol. (2024) 160:1192–200. doi: 10.1001/jamadermatol.2024.3425, PMID: 39356527 PMC11447634

[32] Harman KE Seed PT Gratian MJ Bhogal BS Challacombe SJ Black MM . The severity of cutaneous and oral pemphigus is related to desmoglein 1 and 3 antibody levels. Br J Dermatol. (2001) 144:775–80. doi: 10.1046/j.1365-2133.2001.04132.x, PMID: 11298536

[33] Tsuji-Abe Y Akiyama M Yamanaka Y Kikuchi T Sato-Matsumura KC Shimizu H . Correlation of clinical severity and ELISA indices for the NC16A domain of BP180 measured using BP180 ELISA kit in bullous pemphigoid. J Dermatol Sci. (2005) 37:145–9. doi: 10.1016/j.jdermsci.2004.10.007, PMID: 15734283

[34] Judd KP Lever WF . Correlation of antibodies in skin and serum with disease severity in pemphigus. Arch Dermatol. (1979) 115:428–32. doi: 10.1001/archderm.1979.04010040006002, PMID: 373639

[35] Chorzelski TP Von Weiss JF Lever WF . Clinical significance of autoantibodies in pemphigus. Arch Dermatol. (1966) 93:570–6. doi: 10.1001/archderm.1966.01600230074020 4161271

[36] Beutner EH Jordon RE Chorzelski TP . The immunopathology of pemphigus and bullous pemphigoid. J Invest Dermatol. (1968) 51:63–80. doi: 10.1038/jid.1968.94 4897477

[37] Sams WM Jr. Jordon RE . Correlation of pemphigoid and pemphigus antibody titres with activity of disease. Br J Dermatol. (1971) 84:7–13. doi: 10.1111/j.1365-2133.1971.tb14190.x, PMID: 5573187

[38] Judd KP Mescon H . Comparison of different epithelial substrates useful for indirect immunofluorescence testing of sera from patients with active pemphigus. J Invest Dermatol. (1979) 72:314–6. doi: 10.1111/1523-1747.ep12531752, PMID: 376754

[39] Creswell SN Black MM Bhogal B Skeete MV . Correlation of circulating intercellular antibody titres in pemphigus with disease activity. Clin Exp Dermatol. (1981) 6:477–83. doi: 10.1111/j.1365-2230.1981.tb02338.x, PMID: 7318238

[40] Schmidt E Kasperkiewicz M Joly P . Pemphigus. Lancet. (2019) 394:882–94. doi: 10.1016/S0140-6736(19)31778-7, PMID: 31498102

[41] Feliciani C Caldarola G Kneisel A Podstawa E Pfütze M Pfützner W . IgG autoantibody reactivity against bullous pemphigoid (BP) 180 and BP230 in elderly patients with pruritic dermatoses. Br J Dermatol. (2009) 161:306–12. doi: 10.1111/j.1365-2133.2009.09266.x, PMID: 19485996

[42] Meijer JM Diercks GFH de Lang EWG Pas HH Jonkman MF . Assessment of diagnostic strategy for early recognition of bullous and nonbullous variants of pemphigoid. JAMA Dermatol. (2019) 155:158–65. doi: 10.1001/jamadermatol.2018.4390, PMID: 30624575 PMC6439538

[43] Schmidt T Sitaru C Amber K Hertl M . BP180- and BP230-specific IgG autoantibodies in pruritic disorders of the elderly: a preclinical stage of bullous pemphigoid? Br J Dermatol. (2014) 171:212–9. doi: 10.1111/bjd.12936, PMID: 24601973

