# Correction: Evaluation of a novel chemiluminescent immunoassay for autoantibody detection in pemphigus and bullous pemphigoid

**DOI:** 10.3389/fimmu.2025.1661243

**Published:** 2025-07-21

**Authors:** Na Zhang, Ming Wang, Lili Hou, Yanyu Zhang, Yan Zhao, Luyang Du, Xiaoqing Li, Xia Liu, Hongwei Zhou, Jiaxuan Hao, Jing Tian, Qiuping Yu, Lixia Li, Fangming Cheng, Hai-Zhou Zhou

**Affiliations:** ^1^ Department of Laboratory Diagnostics, The First Affiliated Hospital of Harbin Medical University, Harbin, China; ^2^ Global Academic Department, Shenzhen YHLO Biotech Co., Ltd, Shenzhen, Guangdong, China; ^3^ Dermatology, The First Affiliated Hospital of Harbin Medical University, Harbin, China; ^4^ Reagent R&D Center, Shenzhen YHLO Biotech Co., Ltd, Shenzhen, Guangdong, China

**Keywords:** pemphigus, bullous pemphigoid, desmoglein, BP180, BP230, chemiluminescent immunoassay

Author Ming Wang was erroneously assigned to affiliation “Department of Pathology, University of Cambridge, Cambridge, United Kingdom”. This affiliation has now been removed and affiliation “Global Academic Department, Shenzhen YHLO Biotech Co., Ltd, Shenzhen, Guangdong, China” has now been added for author Ming Wang.

The conflict of interest statement has been correct to:

“Authors MW, LD, QY, LL and FC were employed by Shenzhen YHLO Biotech Co., Ltd.

The remaining authors declare that the research was conducted in the absence of any commercial or financial relationships that could be construed as a potential conflict of interest.”

The original version of this article has been updated.

