# Supplementary material for: Evaluation of a novel chemiluminescent immunoassay for autoantibody detection in pemphigus and bullous pemphigoid
Source: Front Immunol. 2025 Jun 30;16:1576635. doi: 10.3389/fimmu.2025.1576635 (PMC12256432; doi:10.3389/fimmu.2025.1576635)
Supplement: Supplementary file 1 [file DataSheet1.docx]

**Supplementary data to “Evaluation of a Novel Chemiluminescent Immunoassay for Autoantibody Detection in Pemphigus and Bullous Pemphigoid”**

**Supplementary Fig. 1.** The levels of anti-Dsg1 and anti-Dsg3 antibodies in patients with PV and PF


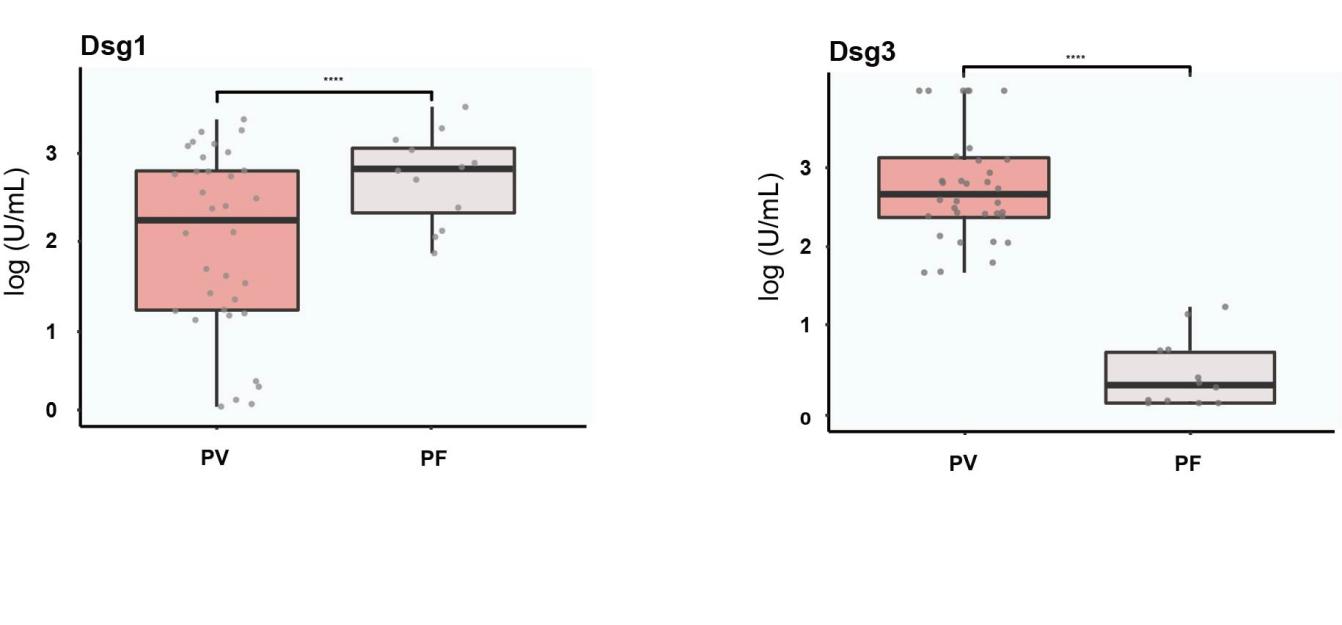


PV, pemphigus vulgaris; PF, pemphigus foliaceus

**Supplementary Fig. 2.** The correlation between anti-Dsg1 and Dsg3 antibodies and the clinical manifestations of patients with PV (Dsg1^-^Dsg3^+^ and Dsg1^+^Dsg3^+^) and PF (Dsg1^+^Dsg3^-^)


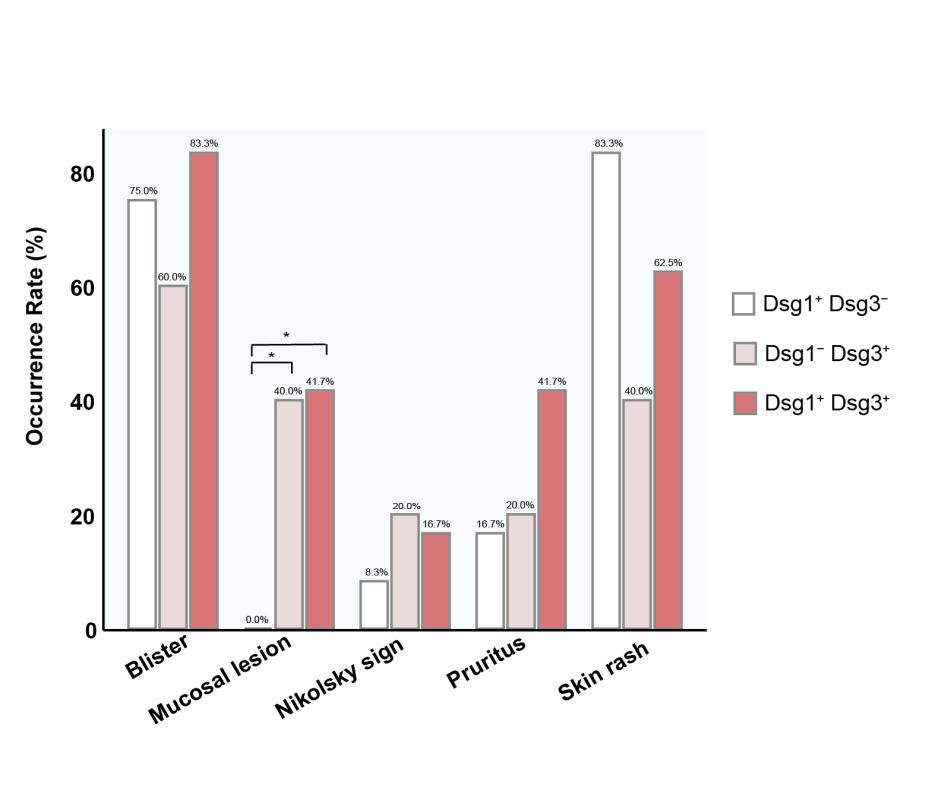


PV, pemphigus vulgaris; PF, pemphigus foliaceus

**Table 1.** Equipments and reagents

| **Instruments** | | | | | | |
| --- | --- | --- | --- | --- | --- | --- |
| **Assay** | | **Equipment Model** | | | **Manufacturer** | |
| CLIA | | iFlash 3000 - C | | | Shenzhen YHLO Biotech Co., Ltd. | |
| ELISA | | PHOMO | | | Zhengzhou Autobio Diagnostics Co., Ltd. | |
| IIFT-BIOCHIP | | Sprinter XL  EUROStar III Plus | | | Euroimmun Medizinische Labordiagnostika AG | |
| **Reagents and Solutions** | | | | | | |
| **Assay** | **Reagent** | | **Components** | | | **Source** |
| CLIA | Anti - Dsg1 (C86223), Anti - Dsg3 (C86224), Anti - BP180 (C86222), Anti - BP230 (C86225) | | Superparamagnetic particles coated with recombinant antigen, along with acridinium - labeled anti - human IgG | | | Shenzhen YHLO Biotech Co., Ltd. |
| ELISA | Anti - Dsg1 (7680E), Anti - Dsg3 (7885E), Anti - BP180 (7695E), Anti - BP230 (7613E) | | Microplates coated with recombinant antigen, enzyme - labeled anti - human IgG, chromogen/substrate solution (TMB/H₂O₂), and stop solution (0.5M sulfuric acid) | | | Euroimmun Medizinische Labordiagnostika AG |
| IIFT-BIOCHIP | Anti - Dsg1, Anti - Dsg3, Anti - BP180, Anti - BP230 (Dermatology Mosaic 60) | | FITC - labeled anti - human IgG, PBS - Tween buffer, coverslips, and mounting medium | | | Euroimmun Medizinische Labordiagnostika AG |
| **Solutions** | | | | | | |
| **Assay** | **Solution** | | | **Preparation Method** | | |
| ELISA | Concentrated Washing Buffer | | | Dilute the concentrated washing buffer with distilled water at a ratio of 1:10 | | |
| IIFT-BIOCHIP | PBS - Tween Buffer | | | Dissolve 1 packet of phosphate in 1 liter of distilled water. Then, add 2 ml of Tween 20 and mix thoroughly | | |

**Supplementary Table 2.** Characteristics of three AIBD autoantibody testing systems.

| Characterization of test reagents for three assays | | | | | | |
| --- | --- | --- | --- | --- | --- | --- |
|  |  | Dsg1 | Dsg3 | BP180 | | BP230 |
| CLIA (YHLO) | Cutoff | P: ≥20 AU/mL | P: ≥20 AU/mL | P: ≥20 AU/mL | | P: ≥20 AU/mL |
|  | Antigen | Recombinant Dsg1 | Recombinant Dsg3 | BP180NC16a | | N-terminus and C-terminus of  BP230 |
| ELISA (MBL) | Cutoff | P: ≥20 UR：≥14, <20 N: ＜14 | P: ≥20 UR：≥7, <20 N: ＜7 | P: ≥9 N: ＜9 | | P: ≥9 N: ＜9 |
|  | Antigen | Recombinant Dsg1 | Recombinant Dsg3 | BP180NC16a | | N-terminus and C-terminus of  BP230 |
| IIFT-BIOCHIP (Euroimmun) | Cutoff | N: ＜1:10 | N: ＜1:10 | N: ＜1:10 | | N: ＜1:10 |
|  | Antigen | Recombinant Dsg1 | Recombinant Dsg3 | BP180-NC16A-4X | | BP230gC |
| Characterization of the detection system for the three assays | | | | | | |
| Feature | CLIA | | ELISA | | IIFT-BIOCHIP | |
| **Automation** | **Fully automated** (integrated pipetting, incubation, washing, detection) | | **Semi-automated or manual** (requires manual pipetting/plate washing) | | **Automatic / Semi-automatic** (microscopic interpretation) | |
| **Assay Time** | 30–60 min (with simultaneous multi-analyte detection per batch) | | 2–3 hours (including multiple incubation/washing steps) | | 2-3 hours (including sample processing/multiple incubation) | |
| **Result Turnaround** | Real-time (direct numerical output by instrument) | | Requires manual absorbance reading and calculation | | Requires experienced personnel for microscopic interpretation (subjective) | |
| **Substrate/**  **Marker** | Magnetic particles + acridinium ester | | Enzyme (HRP/AP) + TMB/H₂O₂ | | Fluorescent-labeled secondary antibody | |
| **Sensitivity** | High (up to pg/mL level) | | Moderate (ng/mL level) | | Moderate (depends on antigen substrate quality) | |
| **Specificity** | High (closed system minimizes cross-reactivity) | | Moderate (potential nonspecific binding) | | High (visualization of tissue/cellular fluorescence patterns) | |
| **Throughput** | High-throughput (hundreds of samples/hour) | | Medium-throughput (depends on plate well capacity) | | Medium/High Throughput (hundreds of samples/hour) | |
| **Primary Applications** | Quantitative detection | | Quantitative/semi-  quantitative screening | | Semiquantitative screening | |

CLIA, chemiluminescence immunoassay; ELISA, enzyme-linked immunosorbent assay; IIFT-BIOCHIP, BIOCHIP mosaic-based indirect immunofluorescence; P, positive; N, negative; UR, Uncertain results.

**Supplementary Table 3.** Reference intervals for AIBD autoantibodies in healthy individuals and in populations with diseases easily confused by AIBD.

| healthy individuals | n | Min | 2.50% | Median | 97.50% | Max |
| --- | --- | --- | --- | --- | --- | --- |
| Dsg1 | 121 | 1 | 1 | 1.31 | 3.35 | 4.54 |
| Dsg3 | 121 | 1 | 1 | 1 | 2.68 | 12.82 |
| BP180 | 121 | 1 | 1 | 1.73 | 5.03 | 160.75 |
| BP230 | 121 | 1 | 1.01 | 1.98 | 5.08 | 10.14 |
| populations with diseases easily confused by AIBD | | | | | | |
| Dsg1 | 153 | 1 | 1 | 1.32 | 4.6725 | 18.1 |
| Dsg3 | 153 | 1 | 1 | 1 | 5.4695 | 8.08 |
| BP180 | 153 | 1 | 1 | 1.81 | 87.5205 | 1881.51 |
| BP230 | 153 | 1 | 1 | 1.805 | 6.9385 | 328.87 |
| healthy individuals and populations with diseases easily confused by AIBD | | | | | | |
| Dsg1 | 274 | 1 | 1 | 1.31 | 4.2415 | 18.1 |
| Dsg3 | 274 | 1 | 1 | 1 | 4.9 | 12.82 |
| BP180 | 274 | 1 | 1 | 1.77 | 26.27 | 1881.51 |
| BP230 | 274 | 1 | 1 | 1.94 | 6.92175 | 328.87 |

**Supplementary Table 4.** Clinical characteristics of AIBD patients.

|  | Dsg1, Median (Q1,Q3) | | | | | | | | Dsg3, Median (Q1,Q3) | | | | | | | | BP180, Median (Q1,Q3) | | | | | | | | BP230, Median (Q1,Q3) | | | | | | | |
| --- | --- | --- | --- | --- | --- | --- | --- | --- | --- | --- | --- | --- | --- | --- | --- | --- | --- | --- | --- | --- | --- | --- | --- | --- | --- | --- | --- | --- | --- | --- | --- | --- |
|  | YES | | NO | | statistic | | p | | YES | | NO | | statistic | | p | | YES | | NO | | statistic | | p | | YES | | NO | | statistic | | p | |
| First diagnosis | 1.48 (1.02, 17.07) | | 1.41 (1.13, 58.03) | | 1880.5 | | 0.749 | | 1.65 (1, 16.24) | | 1.21 (1, 2.86) | | 2163.5 | | 0.341 | | 34.33 (1.97, 587.56) | | 48.47 (1.75, 504.94) | | 2011.5 | | 0.798 | | 2.77 (1.67, 32.76) | | 2.67 (1.67, 6.46) | | 2064.5 | | 0.624 | |
| Skin rash | 1.46 (1, 48.49) | | 1.4 (1.2, 3.08) | | 3140 | | 0.72 | | 1.66 (1, 7.31) | | 1.35 (1, 16.59) | | 3074.5 | | 0.897 | | 37.6 (1.98, 648.55) | | 48.2 (1.9, 512.04) | | 3174 | | 0.635 | | 3.06 (1.43, 33.61) | | 2.36 (1.71, 6.88) | | 3328 | | 0.31 | |
| Blister | 1.43 (1, 26.23) | | 1.44 (1.23, 2.04) | | 2585 | | 0.89 | | 1.69 (1, 13.59) | | 1.14 (1, 5.8) | | 3003 | | 0.14 | | 46.7 (1.97, 667.01) | | 36.11 (1.78, 489.9) | | 2864.5 | | 0.362 | | 2.8 (1.58, 12.64) | | 2.61 (1.68, 26.84) | | 2656.5 | | 0.898 | |
| Pruritus | 1.27 (1, 1.7) | | 2.09 (1.36, 114.78) | | 1788 | | < 0.001 | | 1.54 (1, 3.4) | | 1.81 (1, 138.15) | | 2830 | | 0.224 | | 274.52 (2.92, 702) | | 2.38 (1.71, 97.32) | | 4258 | | < 0.001 | | 3.55 (1.93, 71.33) | | 2.22 (1.59, 3.55) | | 4042 | | 0.003 | |
| Mucosal lesion | 27.25 (1.61, 191.6) | | 1.39 (1, 3.72) | | 1935.5 | | 0.002 | | 315.44(25.21, 1637.75) | | 1.45 (1, 4.83) | | 2083.5 | | < 0.001 | | 2.38 (1.61, 13.53) | | 74.9 (1.99, 600.73) | | 801.5 | | 0.005 | | 2.06 (1.47, 2.96) | | 2.79 (1.67, 35.02) | | 942 | | 0.036 | |
| Nikolsky sign | 134.39 (73.89, 744.97) | | 1.4 (1, 8.03) | | 891 | | 0.003 | | 662.46 (337.62, 5446.12) | | 1.5 (1, 6.24) | | 947 | | < 0.001 | | 1.92 (1.77, 2.2) | | 60.64 (1.98, 591.5) | | 264 | | 0.024 | | 1.76 (1.56, 2.4) | | 2.74 (1.67, 25.98) | | 329.5 | | 0.086 | |
| Severity of lesion | Mild(n = 13) | Mod-erate (n = 9) | | Sever-e (n = 23) | | Stat-istic | | p | Mild(n = 13) | Mod-erate (n = 9) | | Sever-e (n = 23) | | Stat-istic | | p | Mild(n = 2) | Mod-erate (n = 13) | | Sever-e (n = 76) | | Stat-istic | | p | Mild(n = 2) | Mod-erate (n = 13) | | Sever-e(n = 76) | | Stat-istic | | p |
|  | 17.34 (2.87, 134.39) | 237.2 (114.78, 307.57) | | 608.95 (46.48, 1116.72) | | 6.233 | | 0.044 | 116.13 (1.82, 676.68) | 4.7 (1.59, 315.44) | | 278.3 (15.41, 1086.11) | | 2.573 | | 0.276 | 294.73 (192.1, 397.36) | 61.72 (10.13, 667.85) | | 500.98 (43.99, 858.69) | | 1.429 | | 0.489 | 5.38 (3.19, 7.58) | 4.07 (1.23, 46.18) | | 4.4 (2.35, 118.21) | | 1.939 | | 0.379 |

**Supplementary Table 5.** Consistency comparison of IIFT-BIOCHIP and ELISA for the detection of AIBD autoantibodies.

| Consistency of IIFT-BIOCHIP and ELISA for the detection of Dsg1 | | |  |
| --- | --- | --- | --- |
| Dsg1 |  | IIFT-BIOCHIP | 95%CI |
| ELISA | PPA | 50% | 0.41~0.59 |
|  | NPA | 100% | 1~1 |
|  | Consistency values | 87% | 0.80~0.93 |
| Consistency of IIFT-BIOCHIP and ELISA for the detection of Dsg3 | | |  |
| Dsg3 |  | IIFT-BIOCHIP | 95%CI |
| ELISA | PPA | 50% | 0.41~0.59 |
|  | NPA | 100% | 1~1 |
|  | Consistency values | 92% | 0.87~0.97 |
| Consistency of IIFT-BIOCHIP and ELISA for the detection of BP180 | | |  |
| BP180 |  | IIFT-BIOCHIP | 95%CI |
| ELISA | PPA | 82% | 0.75~0.89 |
|  | NPA | 100% | 1~1 |
|  | Consistency values | 86% | 0.80~0.92 |
| Consistency of IIFT-BIOCHIP and ELISA for the detection of BP230 | | |  |
| BP230 |  | IIFT-BIOCHIP | 95%CI |
| ELISA | PPA | 49% | 0.41~0.58 |
|  | NPA | 100% | 1~1 |
|  | Consistency values | 68% | 0.60~0.76 |

PPA, positive percent agreements; NPA, negative percent agreements

**Supplementary Table 6.** Consistency comparison of different methods for the detection of AIBD autoantibodies in patients with pemphigus and bullous pemphigoid.

| Consistency of three methods for the detection of Dsg1 in pemphigus patients | | | | |  |  |
| --- | --- | --- | --- | --- | --- | --- |
| Dsg1 |  | IIFT-BIOCHIP | 95%CI | ELISA | 95%CI | |
| CLIA | PPA | 90% | 0.82~0.98 | 93% | 0.83~1 | |
|  | NPA | 93% | 0.86~1 | 100% | 1~1 | |
|  | Consistency values | 91% | 0.83~0.98 | 95% | 0.86~1 | |
| Consistency of three methods for the detection of Dsg3 in pemphigus patients | | | | |  |  |
| Dsg3 |  | IIFT-BIOCHIP | 95%CI | ELISA | 95%CI | |
| CLIA | PPA | 92% | 0.85~1 | 100% | 1~1 | |
|  | NPA | 100% | 1~1 | 100% | 1~1 | |
|  | Consistency values | 94% | 0.88~1 | 100% | 1~1 | |
| Consistency of the three methods in detecting positivity for either Dsg1 or Dsg3 in pemphigus patients | | | | | | |
| Dsg1 or Dsg3 |  | IIFT-BIOCHIP | 95%CI | ELISA | 95%CI | |
| **CLIA** | PPA | 96% | 0.93~0.99 | 43% | 0.34~0.51 | |
|  | NPA | 99% | 0.98~1 | 100% | 1~1 | |
|  | Consistency values | 98% | 0.96~1 | 81% | 0.74~0.88 | |
| Consistency of three methods for the detection of BP180 in bullous pemphigoid patients | | | | |  |  |
| BP180 |  | IIFT-BIOCHIP | 95%CI | ELISA | 95%CI | |
| CLIA | PPA | 99% | 0.96~1 | 87% | 0.80~0.94 | |
|  | NPA | 92% | 0.87~0.98 | 100% | 1~1 | |
|  | Consistency values | 97% | 0.94~1 | 89% | 0.83~0.95 | |
| Consistency of three methods for the detection of BP230 in bullous pemphigoid patients | | | | |  |  |
| BP230 |  | IIFT-BIOCHIP | 95%CI | ELISA | 95%CI | |
| CLIA | PPA | 92% | 0.87~0.97 | 53% | 0.44~0.63 | |
|  | NPA | 94% | 0.90~0.99 | 97% | 0.94~1 | |
|  | Consistency values | 93% | 0.89~0.98 | 67% | 0.58~0.76 | |
| Consistency of the three methods in detecting positivity for either BP180 or BP230 in bullous pemphigoid patients | | | | | | |
| **BP180 or BP230** |  | IIFT-BIOCHIP | 95%CI | ELISA | 95%CI | |
| **CLIA** | PPA | 100% | 1~1 | 82% | 0.75~0.89 | |
|  | NPA | 88% | 0.83~0.93 | 100% | 1~1 | |
|  | Consistency values | 0.89% | 0.84~0.94 | 85% | 0.79~0.91 | |

PPA, positive percent agreements; NPA, negative percent agreements.

**Supplementary Table 7.** Characterization of AIBD patients with inconsistent CLIA and ELISA testing.

| Dsg1 | positive discordence  (n = 16) | positive concordance  (n = 16) | *p* |
| --- | --- | --- | --- |
| Subtypes, n (%) |  |  | < 0.01 |
| Pemphigus | 3 (18.75) | 11 (68.75) |  |
| bullous pemphigoid | 13 (81.25) | 5 (31.75) |  |
| sex, n (%) |  |  | 0.703 |
| male | 12 (75) | 10 (62) |  |
| female | 4 (25) | 6 (38) |  |
| age, Mean ± SD | 67 ± 9.21 | 60.62 ± 10.16 | 0.073 |
| CLIA, Median (Q1,Q3) | 1.58 (1.28, 2.01) | 617.46 (119.75, 909.08) | < 0.001 |
| ELISA, Median (Q1,Q3) | 27.17 (23.25, 31.95) | 222.45 (129.4, 256.24) | < 0.001 |
| Dsg3 |  |  |  |
| Subtypes, n (%) |  |  | < 0.001 |
| Pemphigus | 0 (0) | 7 (70) |  |
| bullous pemphigoid | 10 (100) | 3 (30) |  |
| sex, n (%) |  |  | 0.65 |
| male | 5 (50) | 7 (70) |  |
| female | 5 (50) | 3 (30) |  |
| age, Mean ± SD | 77 ± 11.32 | 64.6 ± 7.85 | 0.016 |
| CLIA, Median (Q1,Q3) | 2.35 (1.02, 4.64) | 680.7 (521.76, 1183.04) | < 0.001 |
| ELISA, Median (Q1,Q3) | 69.3 ± 43.52 | 137 ± 40.37 | 0.002 |
| BP180 |  |  |  |
| Subtypes, n (%) |  |  | 0.058 |
| Pemphigus | 3 (30) | 4 (4.76) |  |
| bullous pemphigoid | 12 (70) | 80 (95.24) |  |
| sex, n (%) |  |  | 0.624 |
| male | 10 (67) | 47 (56) |  |
| female | 5 (33) | 37 (44) |  |
| age, Mean ± SD | 68.73 ± 8.99 | 70.73 ± 10.45 | 0.448 |
| CLIA, Median (Q1,Q3) | 2.95 (2.09, 5.72) | 553.82 (186.89, 868.87) | < 0.001 |
| ELISA, Median (Q1,Q3) | 21.57 (15.48, 24.69) | 101.68 (70.93, 150.72) | < 0.001 |
| BP230 |  |  |  |
| Subtypes, n (%) |  |  | 0.092 |
| Pemphigus | 5 (12.5) | 1 (2.56) |  |
| bullous pemphigoid | 35 (87.5) | 38 (97.44) |  |
| sex, n (%) |  |  | 0.749 |
| male | 26 (65) | 23 (59) |  |
| female | 14 (35) | 16 (41) |  |
| age, Mean ± SD | 68.15 ± 10.99 | 73.37 ± 10.6 | 0.036 |
| CLIA, Median (Q1,Q3) | 2.77 (1.6, 3.95) | 157.12 (71.33, 301.21) | < 0.001 |
| ELISA, Median (Q1,Q3) | 27.36 (14.99, 41.64) | 147.53 (109.24, 172.71) | < 0.001 |
